# Utilization of NGS technologies to investigate transcriptomic and epigenomic mechanisms in trastuzumab resistance

**DOI:** 10.1038/s41598-019-41672-6

**Published:** 2019-03-26

**Authors:** Miguel Nava, Pranabananda Dutta, Robin Farias-Eisner, Jaydutt V. Vadgama, Yanyuan Wu

**Affiliations:** 1 Division of Cancer Research and Training, Department of Medicine, Charles R. Drew University of Medicine and Science, Los Angeles, USA; 2 Jonsson Comprehensive Cancer Center, David Geffen School of Medicine, University of California at Los Angeles, Los Angeles, CA USA

## Abstract

NGS (Next Generation Sequencing) technologies allows us to determine key gene expression signatures that correlate with resistance (and responsiveness) to anti-cancer therapeutics. We have undertaken a transcriptomic and chromatin immunoprecipitation followed by sequencing (ChIP-seq) approach to describe differences in gene expression and the underlying chromatin landscape between two representative HER2+ cell lines, one of which is sensitive (SKBR3) and the other which is resistant (JIMT1) to trastuzumab. We identified differentially expressed genes (DEGs) and differentially expressed transcripts (DETs) between SKBR3 and JIMT1 cells. Several of the DEGs are components of the Polycomb Repressing Complex 2 (PRC2), and they are expressed higher in JIMT1 cells. In addition, we utilized ChIP-seq to identify H3K18ac, H3K27ac and H3K27me3 histone modifications genome-wide. We identified key differences of H3K18ac and H3K27ac enrichment in regulatory regions, found a correlation between these modifications and differential gene expression and identified a transcription factor binding motif for LRF near these modifications in both cell lines. Lastly, we found a small subset of genes that contain repressive H3K27me3 marks near the gene body in SKBR3 cells but are absent in JIMT1. Taken together, our data suggests that differential gene expression and trastuzumab responsiveness in JIMT1 and SKBR3 is determined by epigenetic mechanisms.

## Introduction

HER2-positive (HER2+) breast cancer accounts for 20–25% of all breast cancers^1^. Prior to the clinical approval of trastuzumab, patients diagnosed with HER2+ breast cancer exhibited the worst prognosis and highest mortality^2^. Monoclonal antibody therapies, such as trastuzumab and pertuzumab, and receptor tyrosine kinase inhibitors, such as Lapatinib, directed against the Human Epidermal Receptors (HER) have vastly improved HER2+ breast cancer patient outcomes^2,3^. Nonetheless, resistance to therapies is a clinical reality. It is estimated that 60–80% of HER2+ breast cancer patients treated with trastuzumab develop resistance^1^.

HER2 is a classical receptor tyrosine kinase (RTK) and its signal transduction potential is realized by heterodimerization with other ligand bound HER family members, such as EGFR/HER1^4–6^. Primary or acquired resistance of HER2+ breast cancer tumors to therapies, including trastuzumab, has been a major challenge for clinical management of this disease. Resistance to trastuzumab involves a myriad of mechanisms including, but not limited to: intrinsic alternations in HER2 receptor (e.g. deletions of the regions coding the trastuzumab binding site), loss of antibody-dependent cell-mediated cytotoxicity (ADCC), intracellular alterations in HER2 downstream signaling, and crosstalk between receptors and signaling pathways leading to activation of other HER family receptors, such as EGFR^7^.

SKBR3 cells were isolated from pleural effusion cells of a Caucasian female patient who had undergone several rounds of treatment with radiation^8^. SKBR3 cells are sensitive to trastuzumab, but trastuzumab resistant SKBR3 cells have been generated by us and others in a laboratory setting^9,10^. We previously demonstrated that SKBR3 (lab generated) trastuzumab-resistant cells expressed higher levels of WNT3 and EGFR than parental cells^9^. JIMT1 cells, which are intrinsically resistant to trastuzumab and are also from pleural effusion cells from a Caucasian female^11^, also expressed higher levels of WNT3 but not EGFR compared to SKBR3 cells^9^ (data not shown). Some groups have conducted comparisons between SKBR3 and JIMT1 cells and have used systems biology approach^12^ which uses established sub-pathway identification and network permutation method. They identified 32 upregulated KEGG sub-pathway genes that were common to trastuzumab resistant cells versus trastuzumab sensitive cells. The network consisted of 4502 sub-pathways. Another excellent review byMartin-Castillo *et al*.^13^ suggest interesting roles for Cancer Stem Cell (CSC) and non-Cancer Stem Cells (non-CSC) within HER2-overexpressing breast carcinomas. They suggest that it is critical to understand the structural interactions between CSC and non-CSC for trastuzumab-based treatment decisions in the clinic. The authors have discussed extensively the biological significance of CSC features and the EMT on the molecular effects and efficacy of trastuzumab in HER2-positive breast cancer cells. They have also focused on the genetic heterogeneity that differentiates trastuzumab-responders from non-responders in terms of CSC cellular states.

In addition to these approaches, we suggest that the chromatin landscape needs to be investigated in these cell lines (SKBR3, JMT1 and others) to determine how the epigenome might contribute to observations made at the level of gene expression. Therefore, we conducted a transcriptomic and ChIP-seq interrogation of JIMT1 and SKBR3 cells to determine differential gene expression and epigenomic differences.

## Results

### Differential gene expression utilizing RNA-seq in JIMT1 and SKBR3 cells

**Figure 1:**
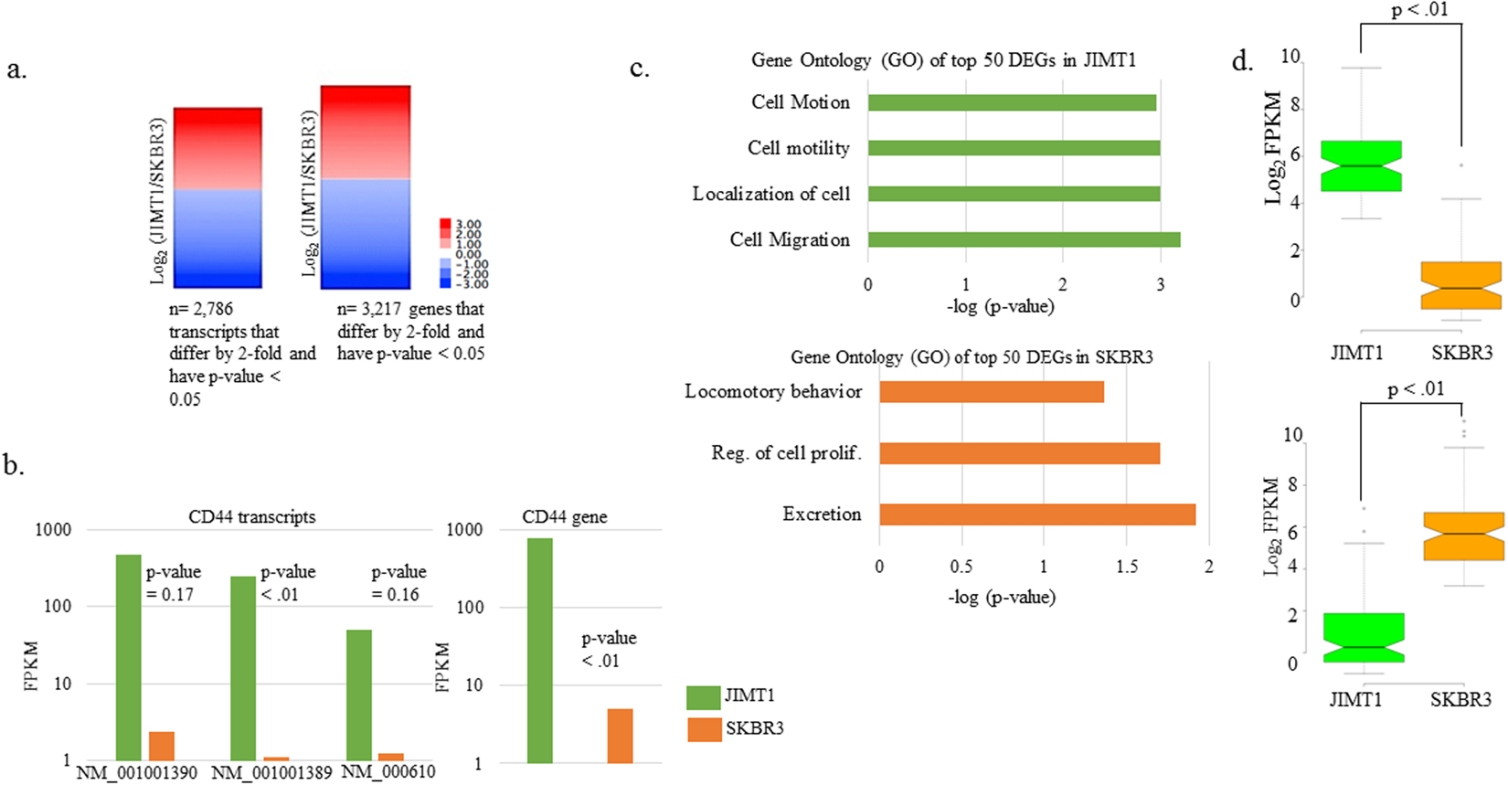
RNA-seq results and Gene Ontology (GO) in top DEGs. (**a**) DETs and DEGs log_2_ ratios of (Fragments Per Kilobase of transcript per Million mapped reads) FPKMs (JIMT1/SKBR3) meeting indicated criteria. Statistical testing was conducted by Cufflinks. (**b**) Three *CD44* DETs that were DE at least 2-fold in JIMT1 relative to SKBR3 cells and their associated p-values as reported by Cufflinks for replicates. *CD44* DE in JIMT1 and SKBR3 cells. (**c**) Gene ontology (GO) terms for top DE genes determined by DAVID^15^. Only p-values (as reported by DAVID) less than 0.05 are shown. (**d**) Two-tailed t-test of top-50 genes shown in (**c)** for each cell line.

We prepared RNA from exponentially growing JIMT1 and SKBR3 cells in biological replicates and conducted RNA-seq. In total, we obtained over 70 million reads that passed quality filters for both replicates (Table 1). Total reads were similar in number between JIMT1 and SKBR3 (Table 1). Only transcripts and genes with an FPKM > 0.5 for both cell lines were analyzed further. We utilized Cufflinks^14^ to determine gene and transcript expression and generated lists containing genes and transcripts that differed by 2-fold between JIMT1 and SKBR3 cells (Supplementary datasets). We added further stringency to our data by also compiling data sets for those genes and transcripts that were differentially expressed (DE) 2-fold or more and had a p-value less than 0.05 (Fig. 1a). In total, over 6000 transcripts were DE 2-fold or more, but only ~2800 passed the statistical threshold (Supplementary datasets). On the other hand, ~4100 genes were DE 2-fold or more and ~3200 of those passed the statistical threshold (Fig. 1a and Supplementary datasets). As an example of one of the well-known differentially expressed genes (DEGs) between JIMT1 and SKBR3 cells, we investigated *CD44* differentially expressed transcripts (DETs)^13^. Three *CD44* transcripts were DE 2-fold or more, but according to Cufflinks, only one of them (NM 001001389) was statistically significant, even though the average difference was 150-fold between JIMT1 and SKBR3 (Fig. 1b). *CD44* gene expression was statistically significant (p-value < 0.01) with 150-fold higher levels in JIMT1 compared to SKBR3 cells (Fig. 1b).

**Table 1:** RNA-seq reads of replicates.

	JIMT1	SKBR3
Rep #1	34,429,857	38,280,766
Rep #2	39,205,022	38,392,607
total reads	73,634,879	76,673,373
passed filters	70,689,484	72,839,704

Reads reported from Illumina HiSeq3000 instrument for replicates and those passing default quality filters.

### Gene ontology (GO) of DEGs between JIMT1 and SKBR3

We determined the GO of the top-50 DEGs with higher expression in JIMT1 using DAVID^15^ (Fig. 1c). On average, gene expression differed ~45-fold between the top-50 DEGs (Fig. 1d). Interestingly, the top-50 DEGs in JIMT1 are involved in cell motion, cell motility and cell migration (Fig. 1c). Examples of these genes includes several of the Annexin gene family, *ANXA1, ANXA8L1* and *ANXA8L2*, as well as genes that encode extracellular matrix functioning proteins, *MSN, KRT5, ITGA6, SERPINE1* and *TIMP1* (Supplementary datasets). The top-50 DEGs with higher expression in SKBR3 are involved in excretion, regulation of cellular proliferation, and locomotory behavior (Fig. 1c). Examples of these genes includes *KRT81, ANXA2, S100A8, S100A9, MUC1, ID2* and *KLF2* (Supplementary datasets). We also determined the most highly expressed genes in each cell line and determined their GO (Supplementary Fig. 1). These genes are different than the DEGs described above and only differed by ~3.5-fold between cell lines, indicating that these genes are highly expressed in both cell lines. The most highly expressed genes in JIMT1 cells are involved in the regulation of metabolism, cytoskeletal organization, and maintenance of protein localization. On the other hand, the most highly expressed genes in SKBR3 cells are involved in metabolism, redox reactions, and nucleus organization (Supplementary Fig. 1). We subjected the DEGs to Gene Set Enrichment Analysis (GSEA) (Supplementary Fig. 2). The analysis showed that 4896/9714 gene sets were upregulated in the JIMT1 phenotype compared to SKBR3. Among those, 257 gene sets were significant at FDR < 0.05 with 513 gene sets significantly enriched at nominal p-value < 0.01 and 1061 gene sets significantly enriched at nominal p-value < 0.05 (Supplementary Table 1).

### Global profiles for H3K18ac and H3K27ac in JIMT1 and SKBR3 cells

In order to investigate the underlying chromatin landscape and to determine whether chromatin regulation could explain differential gene expression between JIMT1 and SKBR3 cells, we conducted ChIP-seq for H3K18ac and H3K27ac. We decided to specifically assay H3K18ac and H3K27ac enrichment because (1) they are marks that are exclusively catalyzed by co-activator paralogs P300/CBP^16^, (2) these marks are enriched in euchromatic regions^17^, (3) these marks increase following activation^18^, and (4) H3K27ac is a marker of super-enhancers^19^. Each ChIP for H3K18ac and H3K27ac replicate had over 20 million reads, but altogether each condition had over 60 million reads that passed quality filters (Table 2).

**Table 2:** ChIP-seq reads of replicates.

Sample	Replicate 1	Replicate 2	Total reads	Passed filters
JIMT1-H3K18ac	31686646	45386845	77073491	75496283
JIMT1-H3K27ac	32606409	28728176	61334585	60451093
JIMT1-H3K27me3	28596918	22941437	51538355	50687288
JIMT1-INPUT	31978471	43889876	75868347	74559927
SKBR3-H3K18ac	28338932	36517693	64856625	63772400
SKBR3-H3K27ac	40314271	23039795	63354066	62490689
SKBR3-H3K27me3	27815082	22296967	50112049	49198653
SKBR3-INPUT	21981986	31279741	53261727	52219799

Reads reported from Illumina HiSeq3000 instrument for replicates and those passing default quality filters.

**Figure 2:**
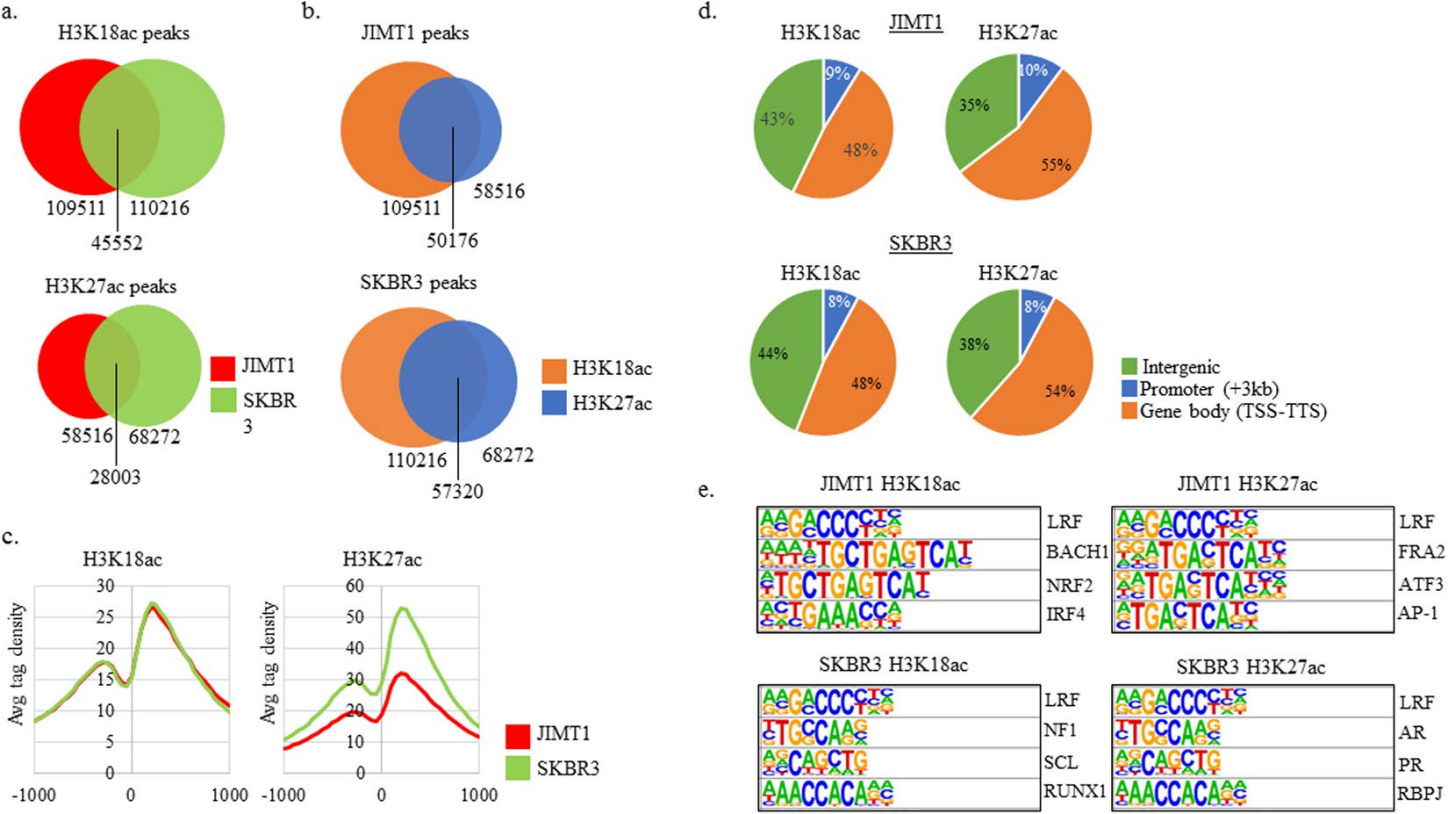
H3K18ac and H3K27ac ChIP-seq and transcription factor binding sites (TFBS) near H3K18ac and H3K27ac. (**a**) Total H3K18ac and H3K27ac peaks in each condition and overlapping peaks between cell lines. (**b**) Comparison of H3K18ac and H3K27ac peaks within the same cell line. (**c**) H3K18ac and H3K27ac peaks at all TSS from −1000 to 1000 as reported by CEAS. **(d**) Locations of H3K18ac and H3K27ac peaks when broken down into three bins: intergenic, promoter (−3kb to TSS) and gene body (TSS to TTS). (**e**) Transcription factor binding sites (TFBS) query results as indicated by HOMER search. All motifs shown were enriched at p-value < 0.01 as reported by HOMER.

JIMT1 and SKBR3 had similar numbers of H3K18ac peaks (Fig. 2a). Approximately 40% of H3K18ac peaks and 45% of H3K27ac were shared between JIMT1 and SKBR3 cells. SKBR3 had ~17% more H3K27ac peaks than JIMT1 cells. The vast majority of H3K18ac and H3K27ac peaks within each cell line overlapped, suggesting that enhancers in JIMT1 and SKBR3 cells contained both modifications (Fig. 2b). Enrichment of H3K18ac near the transcription start site (TSS) of all genes was similar between the cell lines, with peaks at −250 and +200 (Fig. 2c). Interestingly, SKBR3 cells contained higher enrichment of H3K27ac near the TSS of all genes, although the locations of the peaks were similar to the ones observed in the H3K18ac TSS profile (Fig. 2c).

The overall genomic locations of H3K18ac and H3K27ac were similar between JIMT1 and SKBR3 cells when divided into intergenic, promoter (<3 kb upstream from transcription start site TSS) and gene body regions (Fig. 2d). The majority of H3K18ac and H3K27ac peaks in both cell lines were located within gene bodies (Fig. 2d). We determined the transcription factor binding sites (TFBS) that were enriched near H3K18ac and H3K27ac peaks in both cell lines. Interestingly, the motif for LRF (*ZBTB7A*) was enriched in all four conditions. *ZBTB7A*, also known as LRF, was expressed in both cell lines well above the FPKM cutoff (Supplementary Fig. 3). All other enriched motifs were different in ChIP targets and cell lines. Lastly, hormone receptor motifs for progesterone and androgen receptors (PR and AR), were enriched near H3K27ac peaks in SKBR3 cells.

### H3K18ac and H3K27ac at DEGs in JIMT1 and SKBR3 cells

**Figure 3:**
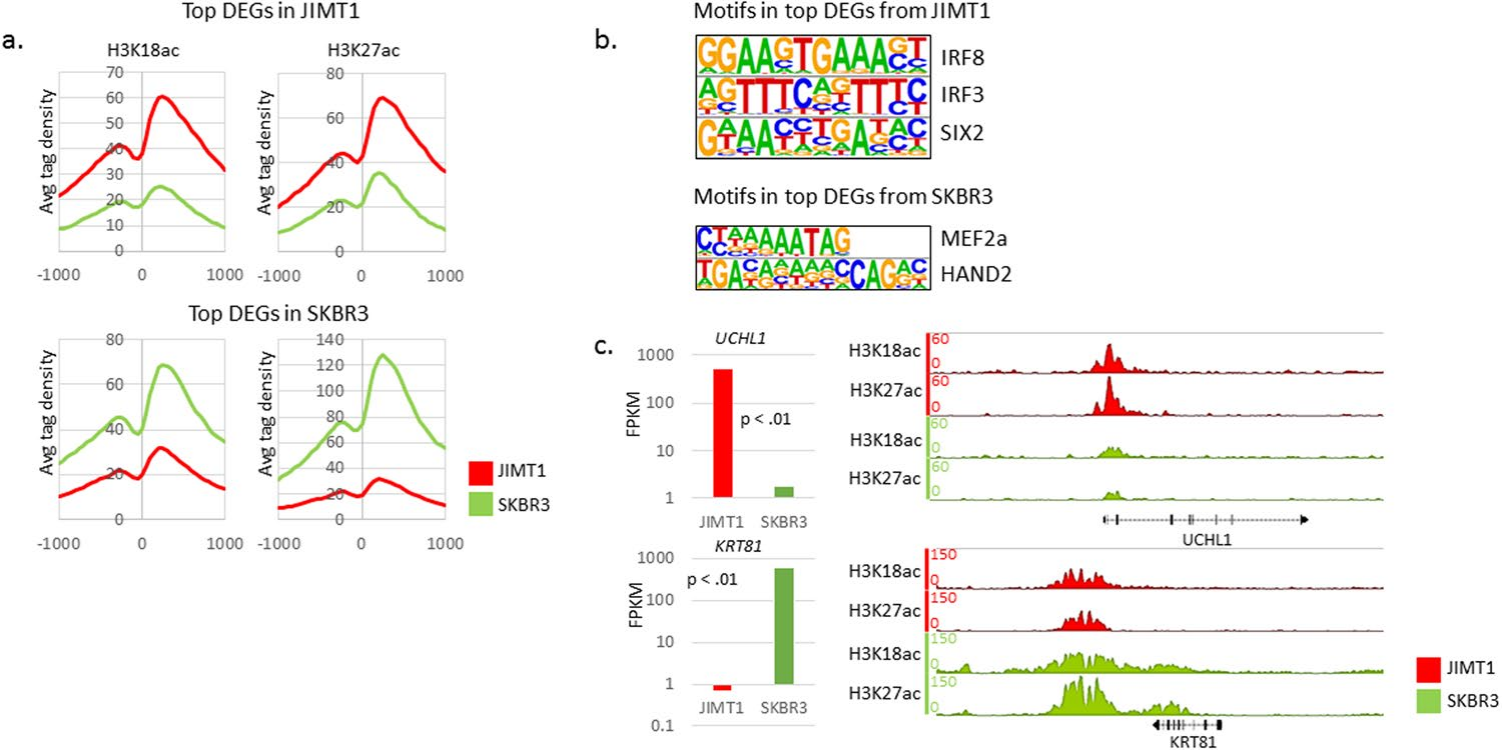
H3K18ac, H3K27ac and TFBS at DEGs. (**a**) H3K18ac and H3K27ac at top-50 DEGs in JIMT1 (top) and SKBR3 (bottom) as reported by CEAS. (**b**) TFBS query results as indicated by HOMER search. All motifs shown were enriched at p-value < 0.01 as reported by HOMER. (**c**) FPKM expression data juxtaposed to IGB ChIP H3K18ac and H3K27ac locus containing gene of interest. *UCHL1* is the top DEG in JIMT1. *KRT81* is the top DEG in SKBR3.

To determine whether the most highly DEGs correlated with chromatin activation marks, we investigated H3K18ac and H3K27ac enrichment centered at the TSS of those genes. The top-250 DEGs in JIMT1 cells contained higher H3K18ac and H3K27ac levels in JIMT1 (Fig. 3a). The top-250 DEGs in SKBR3 cells contained higher H3K18ac and H3K27ac in SKBR3, but the average tag density was vastly greater for H3K27ac throughout the −1000 to +1000 window (Fig. 3a). To gain some insight into putative activators that could be driving expression of the top-250 DEGs in each cell line, we determined the TFBS at the promoters (−300 to +50) of those genes. IRF8, IRF3 and SIX2 binding motifs were enriched in the top-DEGs in JIMT1 cells (Fig. 3b). MEF2a and HAND2 binding motifs were enriched in the top-DEGs in SKBR3 cells. *IRF3* and *SIX2* were more highly expressed in JIMT1 cells and *MEF2a* was more highly expressed in SKBR3 cells (Supplementary Fig. 4).

The most highly DEGs were *UCHL1* and *KRT81* in JIMT1 and SKBR cells, respectively (Supplementary datasets and Fig. 3c). A closer look at H3K18ac and H3K27ac near those genes, illustrates the findings discussed above, namely, higher enrichment of the modifications consistent with differential gene expression. H3K18ac and H3K27ac enrichment was higher in JIMT1 near the *UCHL1* TSS compared to SKBR3 (Fig. 3c). H3K18ac and H3K27ac enrichment was higher in SKBR3 downstream of the *KRT81* transcription termination site and throughout the gene body compared to JIMT1. We investigated H3K18ac and H3K27ac at other DEGs (*ERBB2, CD44* and *CD24*). H3K18ac and H3K27ac enrichment near the loci of those DEGs was correlated to gene expression levels (Supplementary Fig. 5 and data not shown). Taken together, the ChIP-seq data suggests that the differential gene expression between JIMT1 and SKBR cells is determined at the level of chromatin regulation.

### Additional DETs, DEGs and H3K27me3 enrichment in SKBR3 cells

**Figure 4:**
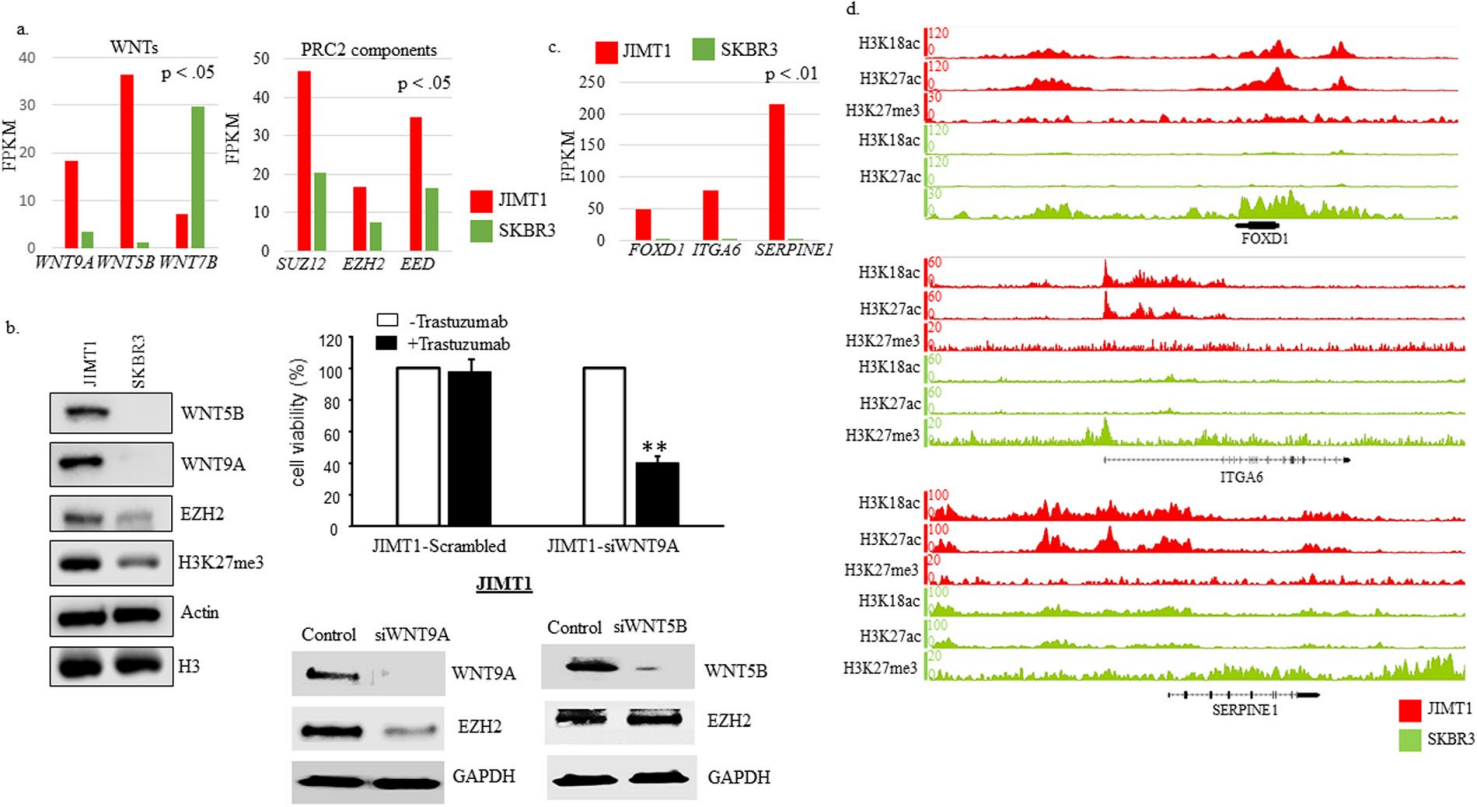
WNTs, PRC2 components, and H3K27me3 enrichment in SKBR3 cells. (**a**) FPKM data for WNTs and PRC2 components in JIMT1 and SKBR3 cells. P-value reported is from Cufflinks output data. (**b**-right) Western blot data comparing whole cell lysates from JIMT1 and SKBR3 cells (n = 2). Western blots utilized same lysates and same gels in most cases, unless samples were overloaded in which case they were rerun with less protein (see Supplementary Fig. 7 for more complete images); (**b**-left top) JIMT1 cells were plated in 96 well plate at 5000 cells/per well and treated with scrambled RNA or siRNA WNT9A for 72 hours. Trastuzumab was added after 24 hours siRNA treatment for 3 days. MTT assay was used for evaluated cell growth. Each data point was 6 measurements and the graph showed mean of 6 measurements plus standard deviation, **p < 0.01 compared to untreated cells; (**b**-right bottom). JIMT1 cells were treated with pooled of two siRNA-sequences against WNT9A (siWNT9A), WNT5B (siWNT5B) or negative sequences (control) for 72 hours and total protein was extracted followed by Western Blot with antibody against WNT9A, WNT5B and EZH2. GAPDH was used as loading control. (**c**) FPKM data for top DEGs in JIMT1, *FOXD1, ITGA6*, and *SERPINE1*, in JIMT1 and SKBR3 cells. P-value reported is from Cufflinks output data. (**d**) IGB screenshots for genes contained in (**c**) containing tracks for H3K18ac, H3K27ac and H3K27me3.

Upon closer investigation of DETs and DEGs, we found that transcripts *SUZ12, EED* and *EZH2* were significantly higher in JIMT1 cells (Fig. 4a). In addition, having previously identified WNT3 as an important molecule in trastuzumab resistance^9^, we searched for WNTs that were DE in the data sets and identified *WNT9A, WNT5B*, and *WNT7B* as DEGs (Fig. 4a). We confirmed differential gene expression for EZH2, SUZ12, WNT5B and WNT9A by subjecting protein lysates from JIMT1 and SKBRB3 to immunoblot analysis (Fig. 4b-left and Supplementary Fig. 6). Interestingly, total H3K27me3 was also higher in JIMT1 cells (Fig. 4b). Wnt/β-catenin signaling has also been shown to play a significant role in both SKBR3 and BT474 cells acquiring resistance that was mainly due to upregulation of WNT3^9^. Knockdown WNT9A by siRNA in JIMT1 showed enhanced JIMT1 cells’ response to trastuzumab. Upon trastuzumab treatment the cell viability was significantly reduced in the WNT9A knockdown cells compared to negative sequence treated cells (JIMT1-Scrambled) (Fig. 4b-right top). Furthermore, knockdown of WNT9A, but not WNT5B in JIMT1 cells decreased EZH2 protein expression (Fig. 4b right-bottom). The data suggests a possible role of PRC2 complexes in trastuzumab resistance that may involve the Wnt signaling pathway. Nonetheless further proof of principle experiments are warranted to validate the involvement of PRC2 in the epigenomic reprogramming of the resistant cells.

SUZ12, EED and EZH2 are components of the Polycomb Repressive Complex-2 (PRC2)^20,21^. PRC2 contains methyltransferase activity, through EZH2, that is responsible for depositing H3K27me3 marks. This modification results in the silencing of chromatin by promoting condensation of the locus^20^. Given the higher expression of *SUZ12*, *EED* and *EZH2* and the higher levels of H3K27me3 in JIMT1, we investigated H3K27me3 by ChIP-seq in JIMT1 and SKBR3 cells (Table 2 and Fig. 4). Surprisingly, inspection of various loci that contained DEGs (*FOXD1, ITGA6, SERPINE1* and *SIX2)* with higher expression in JIMT1 cells, revealed extensive H3K27me3 near or in the coding region in SKBR3 cells but absent in JIMT1 cells (Fig. 4c,d and Supplementary Fig. 7). A locus with several *KRT* genes contained noticeable H3K18ac and H3K27ac peaks but low H3K27me3 peaks in JIMT1 cells. On the other hand, the same region, contained low H3K18ac and H3K27ac, but high H3K27me3 peaks in SKBR3 cells (Supplementary Fig. 7).

### H3K18ac and H3K27ac and Trastuzumab resistance

**Figure 5:**
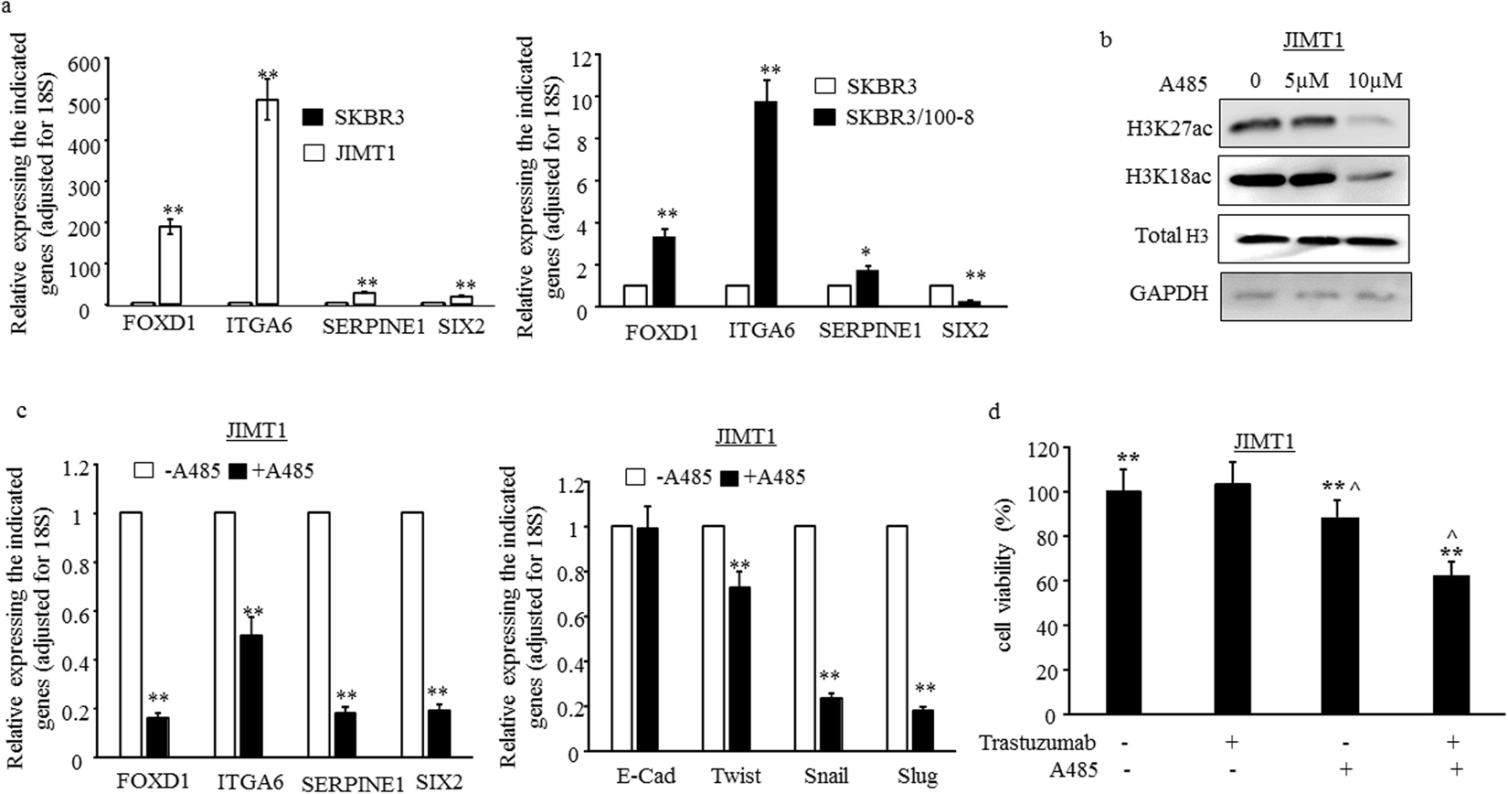
Inhibition of P300/CBP acetylase activity reduces EMT and enhances inhibitory effectiveness of trastuzumab. (**a**) mRNA levels of FOXD1, ITGA6, SERPINE1 and SIX2 were examined by RT-qPCR. The bars indicated mean of three values plus stander deviation. The levels of each indicated genes in SKBR3 were used as references and all levels were adjusted for 18S, *p < 0.05 and **p < 0.01 compared to the levels in SKBR3. (**b**) JIMT1 cells were treated with A-485 for 24 hours and Western blot data comparing whole cell lysates from the A-485 treated and no-treated cells. Antibodies for H3K27ac, H3K18ac, total H3 and GAPDH were used for detecting effect of A-485 in protein levels of H3K27ac and H3K18ac. Total H3 and GAPDH were used as loading control. (**c**) JIMT1 cells were treated with 10 µ of A-485 for 24 hours and mRNA levels of the indicated genes were determined by RT-qPCR. The bars indicated mean of three values plus stander deviation. The levels of each indicated genes in untreated cells were used as references and all levels were adjusted for 18S. **p < 0.01 compared to untreated cells. (**d**) JIMT1 cells were plated in 96 well plate at 5000 cells/per well and treated with (1) 10 µ of A-485, (2) 40 µg trastuzumab, (3) A-485+ trastuzumab, (4) untreated (control) for 72 hours. MTT assay was used for evaluated cell viability. Each data point was 6 measurements and the graph showed mean of 6 measurements plus standard deviation, **p < 0.01 compared to untreated cells.

The higher levels of *FOXD1*, *ITGA6*, *SERPINE1* and *SIX2* in JIMT1 were confirmed by RT-qPCR (Fig. 5a-left panel). To further understand if the higher levels of those genes are associated with trastuzumab resistance, we examined the levels of those genes in another trastuzumab resistant cell line SKBR3/100-8. The SKBR3/100-8 cell line was generated from SKBR3 though clonal selection under trastuzumab exposure^9^. The data showed that the levels of *FOXD1*, *ITGA6* and *SERPINE1* were significantly higher in SKBR3/100-8 compared to parental SKBR3 cells (Fig. 5a-right panel). However, the level of *SIX2* was lower in SKBR3/100-8 compared to SKBR3 (Fig. 5.-right panel). A-485, a selective small-molecule inhibitor of P300/CBP acetylase activity has been shown to decrease H3K18ac and H3K27ac, but not H3K9ac, in prostate cancer cells^22^. Our data showed that A-485 reduced H3K18ac and H3K27ac in JIMT1 cells (Fig. 5b). The levels of *FOXD1*, *ITGA6*, *SERPINE1* and *SIX2* in JIMT1 were reduced significantly following treatment with A-485 (Fig. 5c left-panel). ITGA6 and SIX2 have been indicated promoting EMT (Epithelial-to-mesenchymal transition)^23,24^ and cells undergoing EMT have been shown to be increasingly resistant to trastuzumab^9,25,26^. We found in this study that EMT markers, *TWIST*, *SNAIL* and *SLUG* were also reduced following treatment of JIMT1 cells with A-485 (Fig. 5c right-panel). Moreover, inhibition of P300/CBP in JIMT1 cells reduced cell viability, and the cells showed sensitivity to trastuzumab once treated with a combination of trastuzumab and A-485 (Fig. 5d).

## Discussion

Utilizing NGS methods we conducted a transcriptomic and ChIP-seq based comparison between a trastuzumab sensitive (SKBR3) and resistant (JIMT1) cell line. We determined that there were ~3200 DEGs and ~2800 DETs that were statistically significant (p-value < 0.05) between JIMT1 and SKBR3 cells (Fig. 1a). This greatly expands the number of DEGs and DETs ever reported between JIMT1 and SKBR3 cells^12,13^. As an example, there are eight *CD44* transcripts annotated in hg19, three of these were expressed higher than 2-fold in JIMT1 cells compared to SKBR3 cells (Fig. 1b). However, only one of these three (NM_001001389) was statistically significant due to the variance in the replicates and the limited amount of replicates (n = 2) (data not shown).

By conducting GO analysis of the top-50 DEGs in both cell lines, we determined that the top DEGs in JIMT1 are involved in motility and migration (Fig. 1c). This is consistent with previously published reports that migratory cells, specifically those undergoing Epithelial Mesenchymal Transition (EMT), are more resistant to trastuzumab^25,27^. The most highly DEG was *UCHL1*, expressed over 300-fold higher in JIMT1 cells (Fig. 3c). UCHL1 (Ubiquitin C-terminal hydrolase-L1) has previously been shown to reverse ubiquitylation of HIF-1α and promote metastasis in a HIF-1-dependent manner^28^. Furthermore, overexpression of UCHL1 is consistent with a multi-drug resistance (MDR) phenotype in breast cancer cells and is believed to function through EGFR and MAPK^29,30^. A thorough proteomic investigation could be useful in identifying novel UCHL1 targets that promote HER2+ breast cancer metastasis.

Another interesting DEG that was among the top-50 DEG was *SIX2* (Supplementary Fig. 4). SIX2 is a member of the Sineoculis homeobox homolog family of proteins. All six of the SIX family members have been implicated in breast cancer and *SIX2* is upregulated in basal-like breast cancer^31^. SIX2 promotes metastasis by repressing E-cadherin expression and has also been demonstrated to promote maintenance of a stem cell niche in nephrons through its interaction with TCF/LEF/β-catenin^24,32^. Interestingly, in our data, the SIX2 binding motif was among the most highly enriched in the top-DEGs expressed in JIMT1 cells (Fig. 3b). However, confirmation study showed a lower level of *SIX2* in acquired resistant cells SKBR3/100-8 (Fig. 5a).

We previously identified WNT3 as a molecule that contributed to the development of trastuzumab resistance^9^. Although *WNT3* was expressed over 2-fold higher in JIMT1 cells compared to SKBR3 cells, the FPKM for *WNT3* in SKBR3 was <0.5 and therefore did not make our cutoff (data not shown). However, two other WNTs, *WNT5B* and *WNT9a*, were expressed 32-fold and 5-fold higher, respectively, in JIMT1 cells. (Fig. 4a,b). Knockdown of WNT9a decreased EZH2 and sensitized JIMT1 cells response to trastuzumab (Fig. 4b), suggesting a possible role of PRC2 complexes in trastuzumab resistance and involving Wnt signaling pathway. In this study *WNT5B* made the top-50 DEG list that was utilized in the determination of GO (Fig. 1c). Although WNT5B has been classified as a non-canonical WNT, it activated the canonical WNT pathway in triple negative breast cancer (TNBC) cells^33^. In this context, knockdown of *WNT5B* resulted in decreased cell growth, migration and mammosphere formation. Therefore, WNT5B might also promote trastuzumab resistance and CSC maintenance.

Three Annexin genes, *ANXA1, ANXA8L1* and *ANXA8L2*, were among the top-50 DEGs in JIMT1. ANXA1 was recently described as a strong predictor of trastuzumab resistance, primarily through its activation of AKT signaling, and has also been shown to promote cellular invasion in TNBC cells^34,35^. However, in the previous findings, high ANXA1 levels were partially dependent on decreased ARID1A expression. In our data set *ARID1A* levels were comparable in JIMT1 and SKBR3 cells (data not shown). There is scant literature on *ANXAL1* and *ANXAL2*, especially on how they might relate to oncological phenotypes. More investigations on the highly DE Annexin genes and their function will need to be conducted. Lastly, one of the main transcription factors involved in activating EMT genes, SLUG (*SNAI2*), was expressed much higher in JIMT1 compared to SKBR3 (as has previously been reported^23^) but was excluded from our analysis because the FPKM < 0.5 in SKBR3 cells.

Utilizing ChIP-seq we compared H3K18ac and H3K27ac in JIMT1 and SKBR cells. JIMT1 and SKBR3 cells shared less than 50% of H3K18ac and H3K27ac peaks, indicating different chromatin landscapes (Fig. 2a). It is likely that overlapping peaks occurred over LRF motifs, given the presence of the LRF motif in all the ChIPs (Fig. 2e). LRF (*ZBTB7A*) is an oncogenic transcription factor that is induced by TGF-β1 and is aberrantly expressed in breast cancer tissue^36,37^. A genome-wide analysis of LRF binding has yet to be conducted in HER2+ breast cancer cells. Such an investigation could determine the transcriptional targets of LRF. The remaining top transcription factor binding motifs were different in all conditions, possibly explaining the non-overlapping H3K18ac and H3K27ac peaks (Fig. 2a,e). H3K18ac and H327ac peaks demonstrated significant overlap in each cell line, consistent with the modifications being co-enriched at regulatory regions (Fig. 2b). Taken together, our ChIP-seq data suggests that similar H3K18ac and H3K27ac peaks between JIMT1 and SKBR3 are regulated by LRF recruitment of P300/CBP and dissimilar peaks are regulated by the recruitment of P300/CBP to those sites by various other activators.

By focusing on top-DEGs in each cell line we clearly observed that H3K18ac and H3K27ac enrichment strongly corresponded with expression data (Fig. 3a). *UCHL1* was the most highly DEG in JIMT1 cells and its expression was consistent with H3K18ac and H3K27ac enrichment near the 5′ end of *UCHL1* (Fig. 3c). On the other hand, *KRT81* was the most highly DEG in SKBR3 cells and the *KRT81* gene body contained noticeable levels of H3K18ac and H3K27ac that were absent in JIMT1 cells. The transcription factor binding motifs that were enriched in the top-DEGs in each cell line suggest that IRF8/IRF3/SIX2 are putative drivers of DEGs in JIMT1 cells. Nonetheless, only *IRF3* and *SIX2* levels, and not *IRF8*, were detectable by RNA-seq (Supplementary Fig. 4). However, the upregulation of IRF3 and SIX2 were only confirmed in JIMT1 cells. The SIX2 level was lower in SKBR3/100-8 compared to SKBR3 and IRF3 level was similar between SKBR3/100-8 and SKBR3 cells. We will have to conduct follow-up experiments to determine IRF3 and SIX2 protein levels and their role in activating transcriptional targets in JIMT1 cells.

The higher levels of components of PRC2, *SUZ12, EED* and *EZH2* (Fig. 4a,b and Supplementary Fig. 6), in JIMT1 cells prompted us to investigate H3K27me3 by ChIP-seq. There were ~5 fold more H3K27me3 peaks in JIMT1 cells than in SKBR3 cells (data not shown) and this was reflected in western blot analysis (Fig. 4b). Surprisingly, top DEGs, like *FOXD1*, *ITGA6* and *SERPINE1*, in JIMT1 cells contained low levels of H3K27me3 but high levels in SKBR3 cells (Fig. 4c,d). There are conflicting reports about the role and clinical implications of PRC2 and H3K27me3 levels in breast cancer. High PRC2 levels were observed in TNBC and HER2+ tumors and breast cancer cells^38^. However, H3K27me3 levels were highest in luminal A tumors and not in TNBC and HER2+ tumors. In another study, low levels of PRC2 were found to correlate with poor prognosis^39^. Nonetheless our data in this study showed that downregulation of EZH2, due to *WNT9a* knockdown, sensitized JIMT1 cells to trastuzumab, suggesting that the WNT signaling pathway might play a role in regulation of EZH2 and trastuzumab resistance (Fig. 5a,b). However, further functional studies are required to understand the mechanisms and to validate the involvement of PRC2 in the epigenomic reprogramming of the resistant cells. We plan to determine the role of PRC2 in trastuzumab resistance and breast cancer metastasis.

In addition our study further confirmed that high levels of *FOXD1*, *ITGA6* and *SERPINE1* were associated with HER2-overexpressing breast cancer cells resistant to trastuzumab (Fig. 5a) and they were regulated by acetylation of H3K27 and H3K18 (Fig. 5b,c). Inhibition of P300/CBP acetylase activity promoted responsiveness to trastuzumab and this may be due to the downregulation of *FOXD1*, *ITGA6* and *SERPINE1* and inhibition of EMT (Fig. 5c,d).

**Figure 6:**
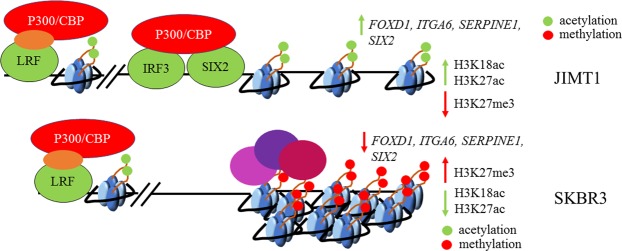
Regulation of gene expression in JIMT1 and SKBR3 cells. IRF/SIX2 motifs were enriched in top-DEGs in JIMT1. These genes contained high levels of H3K18ac and H3K27ac. JIMT1 cells contained low levels of H3K27me3 at these genes. SKBR3 cells contained low levels of H3K18ac and H3K27ac at the same genes. SKBR3 cells contained high levels of H3K27me3 at these genes.

In summary, we propose a model (Fig. 6) Where-upon top-DEGs in JIMT1 are activated by IRF3/SIX2 resulting in recruitment of P300/CBP and subsequent deposition of H3K18ac and H3K27ac marks. This fails to occur in SKBR3 cells because they (1) express lower levels of *IRF3* and *SIX2* and (2) the detection of H3K27me3 near their coding region. Further studies will need to be conducted to test the various characteristics of our model. This includes an investigation of the function of PRC2 in JIMT1 and SKBR3 cells, the genes that are silenced by PRC2 and whether trastuzumab resistance can be overcome by compromising PRC2 function.

## Methods

### RNA-seq

JIMT1 and SKBR3 cells were grown to sub-confluent density. RNA was isolated using Trizol (ThermoFisher Scientific, catalog number 15596026). Nanodrop and Qubit Fluorometric instruments were used to determine RNA concentrations. 1 ug of RNA was used to construct libraries with KAPA mRNA Hyperprep Kit (Roche KK8580). Libraries were sequenced on an Illumina Hiseq3000 instrument.

### RNA-seq analysis

Single end 50 bp mRNA-seq reads were aligned to hg19 using default parameters of Tophat2 (version 2.1.0) and Bowtie2 (version 2.3.2). Samtools (version 0.1.18) was used to convert SAM to BAM files. FPKM values were generated using default parameters for Cufflinks (version 2.1.1). Only FPKM values greater than 0.5 were considered for further analysis. Cufflinks output statistical analysis was utilized to determine significance.

### ChIP-seq

JIMT1 and SKBR3 cells were grown to sub-confluent density. Formaldehyde was added to a final concentration of 1% and incubated for 10 min at 37 °C. Following PBS washing, cells were scraped and washed with 1 mL of PBS containing protease inhibitors (Roche). Cells were resuspended in lysis buffer at a ratio of 5 × 10^6^ cells per 100 ul of lysis buffer. 150 ul of cell lysate was used in chromatin immunoprecipitation with a given antibody. 10 ul of cell lysate was saved for use as input. 3 ug of antibody (H3K18ac (EMD Millipore, catalog 07-354), H3K27ac (Active Motif, catalog 39135) and H3K27me3 (Active Motif, catalog 39155)) was used per ChIP. Following washes and elution, immunoprecipitated material was reverse crosslinked overnight at 65 °C. Samples were treated with RNase A for 30 min at 37 °C and then with Proteinase K for 2 hrs. at 56 °C. DNA was recovered using phenol/chloroform extraction and precipitation. Qubit Fluorometric instrument was used to quantify concentration of recovered DNA. 1 ng of DNA was used to construct libraries with KAPA Hyper Prep Kit (Roche KK8502). Libraries were sequenced on an Illumina Hiseq3000 instrument.

### ChIP-seq analysis

Single end 50 bp reads were aligned to hg19 using default parameters of Bowtie2 (version 2.3.2). Only reads that aligned to a unique position in the genome with no more than two sequence mismatches were retained for further analysis. Peaks were identified with MACS2 (version 2.1.1) using default parameters (p-value < 0.0005). MACS2 output Bedgraph files were converted to BigWig using bedGraphtoBigWig. Next, Bigwig files were converted to Wiggle files for use in CEAS with bigWigtoWig (http://hgdownload.soe.ucsc.edu). Integrated Genome Browser (IGB) was used to view Bedgraph, Bigwig and Wiggle files (http://bioviz.org/igb/). Average H3K18ac and H3K27ac enrichment near the TSS was determined using Cis-regulatory Element Annotation System (CEAS) (http://liulab.dfci.harvard.edu/CEAS/). Bedtools (version 2.26.0) intersect option was used to determine overlapping peaks between H3K18ac or H3K27ac peaks at different time points.

### Transcription factor binding site (TFBS) query

Homer (version 4.9) (homer.ucsd.edu) was used to find enriched motifs from −300 to +50 bp (default settings) of each cluster in the RNA-seq data set. Only motifs that were enriched with a p-value < 0.01 are reported.

### Gene Set Enrichment Analysis (GSEA)

Version 3.0 (http://www.broadinstitute.org/gsea/) was used to conduct GSEA (Broad Institute at MIT, Cambridge, MA) on all DEGs between JIMT1 and SKBR3 cells. GCT and respective CLS file were generated using the FPKM_trackingToGct and ClsFileCreator modules on Genepattern (http://genepattern.broadinstitut.org), respectively. A 2-class analysis with 1000 permutations of gene sets and a weighted metric was used. Given our low number of samples, we utilized the log2 ratio of classes as our metric for ranking genes. Each replicate was considered a different sample (i.e 2 samples per condition (“SKBR3” or “JIMT1”). A false discovery rate (FDR) < 0.05 was considered significant.

### Western blot

Protein lysates were prepared using EBC modified buffer (50 mM Tris-Cl (pH 8.0), 150 mM NaCl, 0.5% NP-40) containing Thermo Scientific Protease and Phosphatase Inhibitor Tablets (A32959). The following antibodies were used: WNT5B (GeneTex, Cat. GTX47174), WNT9A (GeneTex, Cat. GTX128110), EZH2 (Cell Signaling Technology, Cat. 5246S), SUZ12 (Cell Signaling Technology, Cat. 3737), H3 (Cell Signaling Technology, Cat. 5192S) and β-actin (Santa Cruz Biotechnology, sc-69875).

## Supplementary information

Supplementary Figures and Table

Dataset 1

## References

[1] Chung A, Cui X, Audeh W, Giuliano A (2013). Current status of anti-human epidermal growth factor receptor 2 therapies: predicting and overcoming herceptin resistance. Clin Breast Cancer..

[2] Haque R (2012). Impact of breast cancer subtypes and treatment on survival: An analysis spanning two decades. Cancer Epidemiol. Biomarkers Prev..

[3] Vici P (2014). Outcomes of HER2-positive early breast cancer patients in the pre-trastuzumab and trastuzumab eras: a real-world multicenter observational analysis. The RETROHER study. Breast Cancer Res. Treat..

[4] Brennan PJ, Kumogai T, Berezov A, Murali R, Greene MI (2000). HER2/neu: Mechanisms of dimerization/oligomerization. Oncogene.

[5] Rubin I, Yarden Y (2001). The basic biology of HER2. Ann. Oncol..

[6] Yarden Y (2001). The EGFR family and its ligands in human cancer. signalling mechanisms and therapeutic opportunities. Eur. J. Cancer.

[7] Rexer BN, Arteaga CL (2012). Intrinsic and acquired resistance to HER2-targeted therapies in HER2 gene-amplified breast cancer: mechanisms and clinical implications. Crit. Rev. Oncog..

[8] Trempe, G. L. In *Recent results in cancer research. Fortschritte der Krebsforschung. Progres dans les recherches sur le cancer* 33–41 (1976). 10.1007/978-3-642-81043-5_5 1013510

[9] Wu Y (2012). Expression of Wnt3 Activates Wnt/β -Catenin Pathway and Promotes EMT-like Phenotype in Trastuzumab-Resistant HER2-Overexpressing Breast Cancer Cells. Mol. Cancer Res..

[10] Sims JD (2018). Resistance to receptor-blocking therapies primes tumors as targets for HER3-homing nanobiologics. J. Control. Release.

[11] Tanner M (2004). Characterization of a novel cell line established from a patient with Herceptin-resistant breast cancer. Mol. Cancer Ther..

[12] Nam S (2015). A pathway-based approach for identifying biomarkers of tumor progression to trastuzumab-resistant breast cancer. Cancer Lett..

[13] Martin-Castillo B (2013). Basal/HER2 breast carcinomas. Cell Cycle.

[14] Trapnell C (2013). Differential gene and transcript expression analysis of RNA-seq experiments with TopHat and Cufflinks. Nat. Protoc..

[15] Huang, D. W. *et al*. The DAVID Gene Functional Classification Tool: A novel biological module-centric algorithm to functionally analyze large gene lists. *Genome Biol*. **8** (2007). 10.1186/gb-2007-8-9-r183 PMC2375021 17784955

[16] Jin Q (2011). Distinct roles of GCN5/PCAF-mediated H3K9ac and CBP/p300-mediated H3K18/27ac in nuclear receptor transactivation. EMBO J..

[17] Torrente M (2014). Proteomic interrogation of human chromatin protein states. *Syst. Anal. Chromatin-Related Protein Complexes*. Cancer.

[18] Ferrari R (2014). Article Adenovirus Small E1A Employs the Lysine Acetylases p300/CBP and Tumor Suppressor Rb to Repress Select Host Genes and Promote Productive Virus Infection. Cell Host Microbe.

[19] Hnisz, D. *et al*. XSuper-enhancers in the control of cell identity and disease. *Cell* **155** (2013). 10.1016/j.cell.2013.09.053 PMC3841062 24119843

[20] Schuettengruber B, Bourbon HM, Di Croce L, Cavalli G (2017). Genome Regulation by Polycomb and Trithorax: 70 Years and Counting. Cell.

[21] Swigut T, Wysocka J (2007). H3K27 Demethylases, at Long Last. Cell.

[22] Lasko LM (2017). Discovery of a selective catalytic p300/CBP inhibitor that targets lineage-specifi c tumours. Nature.

[23] Zhang K (2017). Oncogenic K-Ras upregulates ITGA6 expression via FOSL1 to induce anoikis resistance and synergizes with αV-Class integrins to promote EMT. Oncogene.

[24] Wang, C. A. *et al*. Homeoprotein Six2 promotes breast cancer metastasis via transcriptional and epigenetic control of E-cadherin expression. *Cancer Res*. 2014 Dec 15, **74**, 7357–7370 (2014). 10.1158/0008-5472.CAN-14-0666 PMC4268359 25348955

[25] Oliveras-Ferraros C (2012). Epithelial-to-mesenchymal transition (EMT) confers primary resistance to trastuzumab (Herceptin). Cell Cycle.

[26] Wu Y (2017). A83-01 inhibits TGF-β-induced upregulation of Wnt3 and epithelial to mesenchymal transition in HER2-overexpressing breast cancer cells. Breast Cancer Res Treat..

[27] May CD (2011). Epithelial-mesenchymal transition and cancer stem cells: a dangerously dynamic duo in breast cancer progression. Breast Cancer Res..

[28] Goto Y (2015). UCHL1 provides diagnostic and antimetastatic strategies due to its deubiquitinating effect on HIF-1α. Nat. Commun..

[29] Jin Y (2015). UCH-L1 involved in regulating the degradation of EGFR and promoting malignant properties in drug-resistant breast cancer. Int. J. Clin. Exp. Pathol..

[30] Wang W (2016). Overexpression of ubiquitin carboxyl terminal hydrolase-L1 enhances multidrug resistance and invasion/metastasis in breast cancer by activating the MAPK/Erk signaling pathway. Mol. Carcinog..

[31] Xu HX (2016). Expression profile of SIX family members correlates with clinic-pathological features and prognosis of breast cancer: A systematic review and meta-analysis. Medicine (Baltimore)..

[32] Park, J. S. *et al*. NIH Public Access. **23**, 637–651 (2012).

[33] Yang L (2014). Wnt modulates MCL1 to control cell survival in triple negative breast cancer. BMC Cancer.

[34] Berns K (2016). Loss of ARID1A activates ANXA1, which serves as a predictive biomarker for trastuzumab resistance. Clin. Cancer Res..

[35] Okano M (2015). Upregulated Annexin A1 promotes cellular invasion in triple-negative breast cancer. Oncol. Rep..

[36] Shen, Y. *et al*. Negative feedback loop between ZBTB7A and TGF - β in breast cancer. 1403–1410, 10.3892/ol.2017.6291 (2017). 10.3892/ol.2017.6291 PMC5529933 28789356

[37] Aggarwal, A. *et al*. NIH Public Access. **89**, 140–148 (2010).

[38] Holm K (2012). Global H3K27 trimethylation and EZH2 abundance in breast tumor subtypes. Mol. Oncol..

[39] Wassef M (2015). Impaired PRC2 activity promotes transcriptional instability and favors breast tumorigenesis. Genes Dev..

