# Supplementary material for: Utilization of NGS technologies to investigate transcriptomic and epigenomic mechanisms in trastuzumab resistance
Source: Sci Rep. 2019 Mar 26;9:5141. doi: 10.1038/s41598-019-41672-6 (PMC6435657; doi:10.1038/s41598-019-41672-6)
Supplement: Supplementary file 1 — Supplementary Figures and Table [file 41598_2019_41672_MOESM1_ESM.docx]

**Title: Utilization of NGS technologies to investigate transcriptomic and epigenomic mechanisms in trastuzumab resistance**

**Authors:** Miguel Nava^1^, Pranabananda Dutta^1^, Robin Farias-Eisner^2^, Jaydutt V. Vadgama*^1, 2^, and Yanyuan Wu*^1, 2^

**Authors’ Affiliations:**

1. Division of Cancer Research and Training, Department of Medicine, Charles R. Drew University of Medicine and Science,
2. Jonsson Comprehensive Cancer Center, David Geffen School of Medicine, University of California at Los Angeles, Los Angeles, CA, USA.

**Authors’ E-mail address:**

MN: [miggy@g.ucla.edu](mailto:miggy@g.ucla.edu)

PD: pranabandutta@cdrewu.edu

RF: [RFEisner@mednet.ucla.edu](mailto:RFEisner@mednet.ucla.edu)

JV: [jayvadgama@cdrewu.edu](mailto:jayvadgama@cdrewu.edu)

YW: [yanyuanwu@cdrewu.edu](mailto:yanyuanwu@cdrewu.edu)

***Corresponding authors:** Dr. Yanyuan Wu, Dr. Jaydutt Vadgama. Division of Cancer Research and Training, Charles R. Drew University of Medicine and Science, 1748 East 120th Street, Los Angeles, CA 90059, USA. Tel: 323-563-4853; Fax: 323-563--4859. Email: [yanyuanwu@cdrewu.edu](mailto:yanyuanwu@cdrewu.edu); [jayvadgama@cdrewu.edu](mailto:jayvadgama@cdrewu.edu).

**Supplementary Figures and Table**

**Supplementary Figure 1. GO of most highly expressed genes. A.** GO terms for most highly expressed genes in each cell line as determined by DAVID. **B.** Two-tailed t-test of most highly expressed genes in each cell line.

**Supplementary Figure 2. GSEA enrichment plots of the DEGs in JIMT1.** Plots were generated by the GSEA program as described in the methods section. Only the top-20 plots for gene sets that were significant at FDR < 0.05 are shown (please see plots associated with GSEA results). **Supplementary Table 1.** **Summary of Enrichment in JIMT1 phenotype**

**Supplementary Figure 3. Expression of *ZBTB7A* in JIMT1 and SKBR3 cells.** FPKM values for *ZBTB7A* as reported by Cufflinks.

**Supplementary Figure 4. Expression of *IRF3, SIX2* and *MEF2a* in JIMT1 and SKBR3 cells.** FPKM values for *IRF3, SIX2* and *MEF2a* as reported by Cufflinks.

**Supplementary Figure 5. H3K18ac and H3K27ac at DEGs.** IGB screenshots for DEGs containing H3K18ac and H3K27ac tracks.

**Supplementary Figure 6. Western blot of SUZ12 in JIMT1 and SKBR3 cells.** Western blot data comparing whole cell lysates from JIMT1 and SKBR3 cells (n=2). Lysates were run on same gel and western blotted for indicated proteins. Please see Supplementary Figure 7 for expanded western.

**Supplementary Figure 7. Additional loci demonstrating H3K18ac, H3K27ac and H3K27me3.** IGB screenshots for DEGs containing H3K18ac, H3K27ac and H3K27me3 tracks.

**Supplementary Figure 8. Expanded western blot results of various gels.** Expanded images of western blot results shown in Figure 4 and Supplementary Figure 5. Asterisks (*) indicate protein band of interest for given western blot.

**Figure S1**


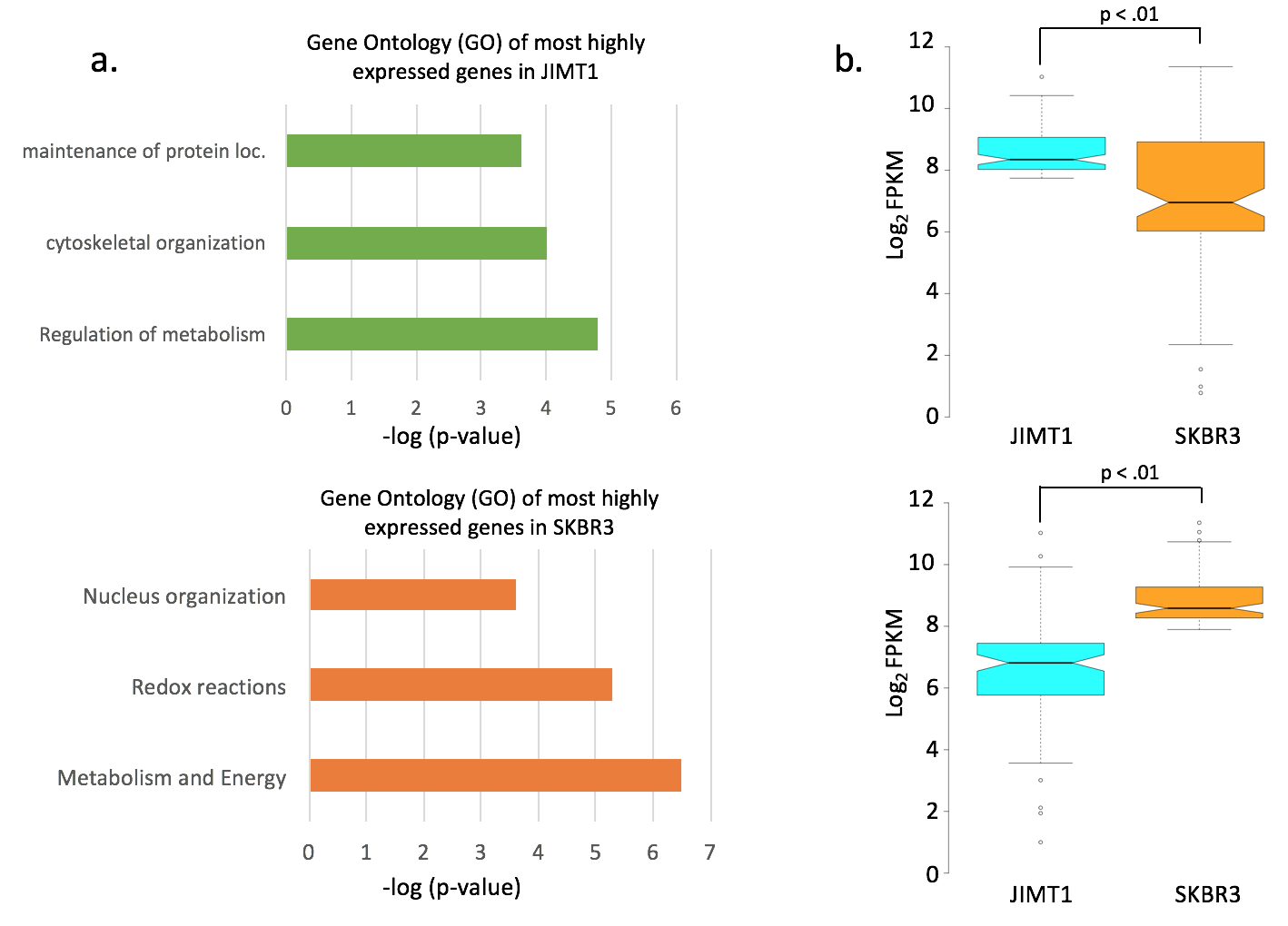


**Figure S2**

**
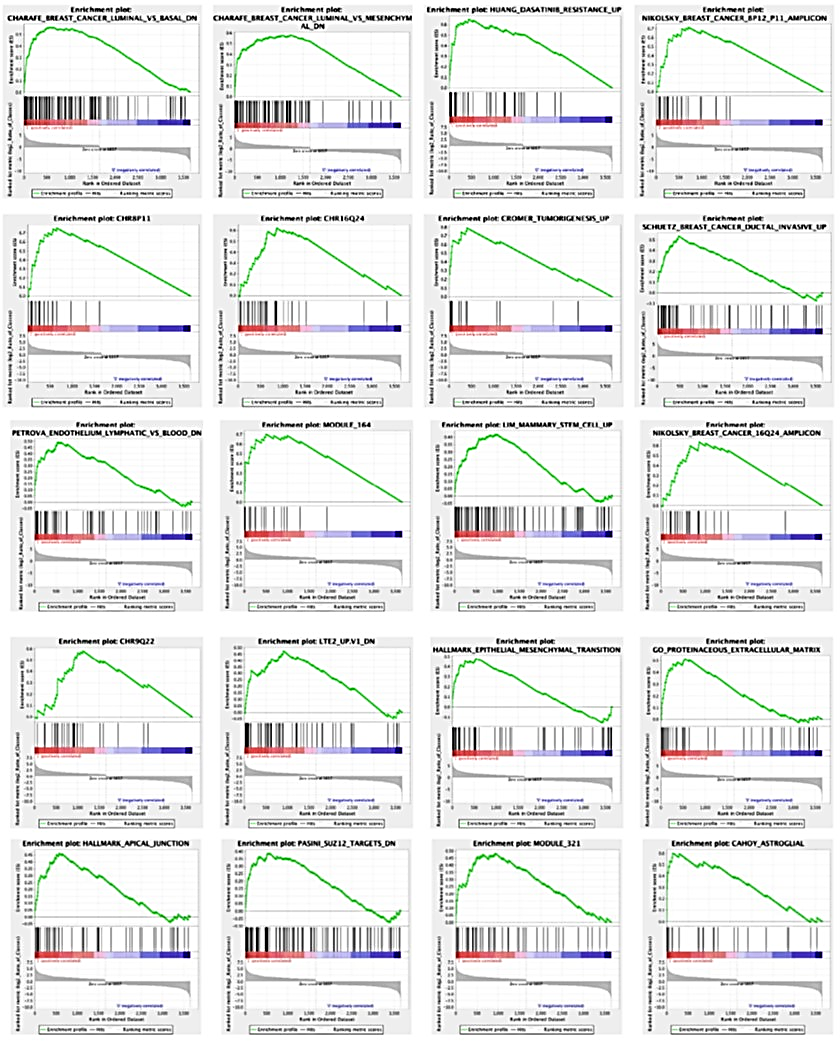
**

**Table S1 Summary of Enrichment in JIMT1 phenotype**

| **NAME** | **ES** | **NES** |
| --- | --- | --- |
| CHARAFE_BREAST_CANCER_LUMINAL_VS_BASAL_DN | 0.5654044 | 4.0920877 |
| CHARAFE_BREAST_CANCER_LUMINAL_VS_MESENCHYMAL_DN | 0.58005327 | 4.0444107 |
| HUANG_DASATINIB_RESISTANCE_UP | 0.6526693 | 3.4159188 |
| NIKOLSKY_BREAST_CANCER_8P12_P11_AMPLICON | 0.71755433 | 3.225395 |
| CHR8P11 | 0.7567768 | 3.124303 |
| CHR16Q24 | 0.6235026 | 3.0808003 |
| CROMER_TUMORIGENESIS_UP | 0.78939366 | 3.0337038 |
| SCHUETZ_BREAST_CANCER_DUCTAL_INVASIVE_UP | 0.53765094 | 3.0124693 |
| PETROVA_ENDOTHELIUM_LYMPHATIC_VS_BLOOD_DN | 0.49409002 | 2.854957 |
| MODULE_164 | 0.70335627 | 2.7812717 |
| LIM_MAMMARY_STEM_CELL_UP | 0.42150006 | 2.7614098 |
| NIKOLSKY_BREAST_CANCER_16Q24_AMPLICON | 0.63581455 | 2.7388828 |
| CHR9Q22 | 0.5834594 | 2.7306557 |
| LTE2_UP.V1_DN | 0.4758377 | 2.7121656 |
| HALLMARK_EPITHELIAL_MESENCHYMAL_TRANSITION | 0.4791071 | 2.67626 |
| GO_PROTEINACEOUS_EXTRACELLULAR_MATRIX | 0.5187953 | 2.667896 |
| HALLMARK_APICAL_JUNCTION | 0.46479625 | 2.6380103 |
| PASINI_SUZ12_TARGETS_DN | 0.3897317 | 2.6335068 |
| MODULE_321 | 0.4864432 | 2.6225314 |
| CAHOY_ASTROGLIAL | 0.60404456 | 2.6195822 |
| GO_NEGATIVE_REGULATION_OF_IMMUNE_RESPONSE | 0.6500812 | 2.6186943 |
| BASAKI_YBX1_TARGETS_UP | 0.3904726 | 2.5836623 |
| GO_REGULATION_OF_CELL_SUBSTRATE_ADHESION | 0.48628107 | 2.5737422 |
| GSE30971_WBP7_HET_VS_KO_MACROPHAGE_4H_LPS_STIM_UP | 0.48132634 | 2.5595393 |
| DER_IFN_BETA_RESPONSE_UP | 0.5187657 | 2.5407145 |
| PETROVA_PROX1_TARGETS_DN | 0.6297143 | 2.531899 |
| HOELZEL_NF1_TARGETS_UP | 0.5280124 | 2.5292168 |
| GO_NEGATIVE_REGULATION_OF_IMMUNE_EFFECTOR_PROCESS | 0.62645924 | 2.5280077 |
| MODULE_357 | 0.5103492 | 2.5210521 |
| GO_EXTRACELLULAR_MATRIX | 0.43125835 | 2.5144038 |
| KOBAYASHI_EGFR_SIGNALING_24HR_DN | 0.4244095 | 2.503074 |
| GO_NEGATIVE_REGULATION_OF_PEPTIDASE_ACTIVITY | 0.5095251 | 2.4971623 |
| MODULE_297 | 0.5238987 | 2.4933333 |
| CHICAS_RB1_TARGETS_CONFLUENT | 0.33678848 | 2.4817724 |
| GO_REGULATION_OF_CELL_MATRIX_ADHESION | 0.5440529 | 2.473154 |
| NAKAMURA_TUMOR_ZONE_PERIPHERAL_VS_CENTRAL_UP | 0.42812663 | 2.459445 |
| GO_NEGATIVE_REGULATION_OF_CELL_CELL_ADHESION | 0.5479729 | 2.4572918 |
| WALLACE_PROSTATE_CANCER_RACE_UP | 0.44777504 | 2.4431975 |
| GO_PEPTIDASE_REGULATOR_ACTIVITY | 0.53418094 | 2.442253 |
| MODULE_154 | 0.52948743 | 2.4420772 |
| HENDRICKS_SMARCA4_TARGETS_UP | 0.5723482 | 2.4412901 |
| STAMBOLSKY_TARGETS_OF_MUTATED_TP53_DN | 0.5773997 | 2.4398656 |
| WU_CELL_MIGRATION | 0.39146987 | 2.4383588 |
| KEGG_FOCAL_ADHESION | 0.43444836 | 2.4215438 |
| CHR8Q22 | 0.54007137 | 2.409546 |
| BERENJENO_TRANSFORMED_BY_RHOA_UP | 0.34823605 | 2.4072828 |
| PEDERSEN_TARGETS_OF_611CTF_ISOFORM_OF_ERBB2 | 0.5106835 | 2.4058642 |
| SANA_TNF_SIGNALING_UP | 0.60102063 | 2.404049 |
| CHR12Q24 | 0.43801543 | 2.4004052 |
| REN_ALVEOLAR_RHABDOMYOSARCOMA_DN | 0.34597793 | 2.3957152 |
| SMID_BREAST_CANCER_LUMINAL_B_DN | 0.36149162 | 2.3876278 |
| SERVITJA_ISLET_HNF1A_TARGETS_UP | 0.5254277 | 2.3768911 |
| GO_EXTRACELLULAR_STRUCTURE_ORGANIZATION | 0.4186266 | 2.3757637 |
| GO_PEPTIDASE_INHIBITOR_ACTIVITY | 0.5892501 | 2.3616712 |
| GO_REGULATION_OF_CELL_ADHESION | 0.33097363 | 2.3500867 |
| KEGG_ECM_RECEPTOR_INTERACTION | 0.6480474 | 2.3485816 |
| ESC_V6.5_UP_EARLY.V1_DN | 0.4133786 | 2.3484228 |
| MODULE_3 | 0.33933643 | 2.3452728 |
| REACTOME_INTERFERON_GAMMA_SIGNALING | 0.5973193 | 2.3424158 |
| BRUECKNER_TARGETS_OF_MIRLET7A3_DN | 0.5401323 | 2.3373718 |
| DAVICIONI_PAX_FOXO1_SIGNATURE_IN_ARMS_UP | 0.57985306 | 2.3325703 |
| PID_INTEGRIN1_PATHWAY | 0.61801356 | 2.328325 |
| WATANABE_RECTAL_CANCER_RADIOTHERAPY_RESPONSIVE_DN | 0.5018297 | 2.3220208 |
| GO_NEGATIVE_REGULATION_OF_EPITHELIAL_CELL_PROLIFERATION | 0.516557 | 2.3087733 |
| GSE15750_DAY6_VS_DAY10_EFF_CD8_TCELL_UP | 0.4107642 | 2.3074174 |
| GSE29618_PDC_VS_MDC_DAY7_FLU_VACCINE_DN | 0.40061337 | 2.305016 |
| RODWELL_AGING_KIDNEY_UP | 0.35151407 | 2.3042798 |
| CHRXP11 | 0.5206343 | 2.3034053 |
| GSE42021_CD24HI_VS_CD24LOW_TREG_THYMUS_DN | 0.43737325 | 2.3026795 |
| LABBE_TARGETS_OF_TGFB1_AND_WNT3A_UP | 0.4870604 | 2.3023498 |
| MIYAGAWA_TARGETS_OF_EWSR1_ETS_FUSIONS_DN | 0.41920748 | 2.3011014 |
| PICCALUGA_ANGIOIMMUNOBLASTIC_LYMPHOMA_UP | 0.4765205 | 2.289043 |
| GO_NEGATIVE_REGULATION_OF_CELL_ADHESION | 0.41837144 | 2.2885969 |
| GRUETZMANN_PANCREATIC_CANCER_UP | 0.34694856 | 2.2858377 |
| GSE14415_ACT_TCONV_VS_ACT_NATURAL_TREG_DN | 0.41404358 | 2.2821171 |
| CHR3Q13 | 0.6044132 | 2.281507 |
| WANG_METHYLATED_IN_BREAST_CANCER | 0.55744463 | 2.2755775 |
| ERB2_UP.V1_DN | 0.4050365 | 2.271514 |
| GOLDRATH_NAIVE_VS_MEMORY_CD8_TCELL_DN | 0.38783568 | 2.2704446 |
| AMIT_EGF_RESPONSE_480_HELA | 0.4125318 | 2.2620816 |
| GSE41978_ID2_KO_VS_BIM_KO_KLRG1_LOW_EFFECTOR_CD8_TCELL_UP | 0.4030522 | 2.2603905 |
| GSE40068_CXCR5NEG_BCL6NEG_CD4_TCELL_VS_CXCR5POS_BCL6NEG_TFH_DN | 0.3948386 | 2.2583876 |
| AMIT_SERUM_RESPONSE_240_MCF10A | 0.538095 | 2.256685 |
| GO_POSITIVE_REGULATION_OF_LOCOMOTION | 0.33049846 | 2.25208 |
| GSE21546_UNSTIM_VS_ANTI_CD3_STIM_SAP1A_KO_DP_THYMOCYTES_UP | 0.3788836 | 2.2517302 |
| GO_MEMBRANE_MICRODOMAIN | 0.35893098 | 2.2507632 |
| KATSANOU_ELAVL1_TARGETS_DN | 0.46166614 | 2.24754 |
| FUJII_YBX1_TARGETS_DN | 0.4018912 | 2.2452307 |
| PEDERSEN_METASTASIS_BY_ERBB2_ISOFORM_4 | 0.44601035 | 2.2447894 |
| BROWNE_HCMV_INFECTION_2HR_DN | 0.5646705 | 2.2430573 |
| XU_HGF_SIGNALING_NOT_VIA_AKT1_48HR_UP | 0.5923633 | 2.2428286 |
| WIEDERSCHAIN_TARGETS_OF_BMI1_AND_PCGF2 | 0.5090072 | 2.2347546 |
| GSE18804_SPLEEN_MACROPHAGE_VS_TUMORAL_MACROPHAGE_DN | 0.3836416 | 2.234677 |
| RODRIGUES_THYROID_CARCINOMA_DN | 0.4861103 | 2.2321901 |
| CHR16Q22 | 0.4720026 | 2.2305872 |
| GSE14000_UNSTIM_VS_16H_LPS_DC_DN | 0.38048443 | 2.2287645 |
| PHONG_TNF_RESPONSE_NOT_VIA_P38 | 0.33175325 | 2.2249374 |
| GO_NEGATIVE_REGULATION_OF_HOMOTYPIC_CELL_CELL_ADHESION | 0.54689485 | 2.2171059 |
| GO_NEGATIVE_REGULATION_OF_PROTEOLYSIS | 0.40176842 | 2.2132807 |
| KEGG_REGULATION_OF_ACTIN_CYTOSKELETON | 0.39537174 | 2.2090416 |
| RUIZ_TNC_TARGETS_DN | 0.43054405 | 2.208673 |
| GO_REGULATION_OF_CELL_JUNCTION_ASSEMBLY | 0.53370565 | 2.2084277 |
| GO_CILIUM_MORPHOGENESIS | 0.3811239 | 2.204145 |
| GO_REGULATION_OF_ADAPTIVE_IMMUNE_RESPONSE | 0.5226779 | 2.2026894 |
| GOLDRATH_ANTIGEN_RESPONSE | 0.33869734 | 2.202203 |
| GO_POSITIVE_REGULATION_OF_CELL_ADHESION | 0.35152325 | 2.1891396 |
| KIM_WT1_TARGETS_8HR_UP | 0.39432672 | 2.1878633 |
| GSE13547_2H_VS_12_H_ANTI_IGM_STIM_BCELL_DN | 0.4117488 | 2.1824646 |
| JOHNSTONE_PARVB_TARGETS_2_UP | 0.41210973 | 2.1752493 |
| TAKEDA_TARGETS_OF_NUP98_HOXA9_FUSION_3D_UP | 0.444839 | 2.175221 |
| GO_SIDE_OF_MEMBRANE | 0.36542255 | 2.1740944 |
| KEGG_SMALL_CELL_LUNG_CANCER | 0.50547963 | 2.1672127 |
| GO_REGULATION_OF_CELLULAR_COMPONENT_MOVEMENT | 0.29721883 | 2.165581 |
| GSE13522_WT_VS_IFNAR_KO_SKING_T_CRUZI_Y_STRAIN_INF_DN | 0.43995395 | 2.1638598 |
| CREIGHTON_ENDOCRINE_THERAPY_RESISTANCE_2 | 0.33690247 | 2.1580179 |
| GO_BASEMENT_MEMBRANE | 0.49516243 | 2.156658 |
| GSE14415_INDUCED_TREG_VS_FAILED_INDUCED_TREG_UP | 0.40203145 | 2.1551168 |
| COLDREN_GEFITINIB_RESISTANCE_UP | 0.49766606 | 2.150715 |
| GSE11057_NAIVE_VS_MEMORY_CD4_TCELL_DN | 0.35509014 | 2.1491501 |
| ICHIBA_GRAFT_VERSUS_HOST_DISEASE_D7_UP | 0.5030996 | 2.1454232 |
| VERHAAK_GLIOBLASTOMA_MESENCHYMAL | 0.36105576 | 2.1434047 |
| GSE36888_UNTREATED_VS_IL2_TREATED_STAT5_AB_KNOCKIN_TCELL_2H_UP | 0.37921497 | 2.1410663 |
| DER_IFN_GAMMA_RESPONSE_UP | 0.4781503 | 2.1400516 |
| GO_NEGATIVE_REGULATION_OF_DEFENSE_RESPONSE | 0.448036 | 2.1388032 |
| BMI1_DN.V1_UP | 0.40112403 | 2.138692 |
| GO_PLASMA_MEMBRANE_RECEPTOR_COMPLEX | 0.4622947 | 2.1384037 |
| SENESE_HDAC1_AND_HDAC2_TARGETS_UP | 0.3856537 | 2.1368198 |
| SMID_BREAST_CANCER_BASAL_UP | 0.30359775 | 2.1359901 |
| GO_CELL_SUBSTRATE_JUNCTION | 0.3061692 | 2.134833 |
| LANDIS_BREAST_CANCER_PROGRESSION_DN | 0.54534316 | 2.131621 |
| DER_IFN_ALPHA_RESPONSE_UP | 0.46283844 | 2.1288908 |
| GSE1740_UNSTIM_VS_IFNA_STIMULATED_MCSF_DERIVED_MACROPHAGE_DN | 0.41047668 | 2.1280644 |
| GSE29618_MONOCYTE_VS_PDC_DAY7_FLU_VACCINE_UP | 0.41257498 | 2.1237042 |
| GO_NEGATIVE_REGULATION_OF_RESPONSE_TO_WOUNDING | 0.42590842 | 2.1227803 |
| CHR6P21 | 0.3968146 | 2.117709 |
| GO_POSITIVE_REGULATION_OF_ADAPTIVE_IMMUNE_RESPONSE | 0.5617319 | 2.11698 |
| CHR10Q24 | 0.5054721 | 2.1169698 |
| GSE15750_DAY6_VS_DAY10_TRAF6KO_EFF_CD8_TCELL_UP | 0.38579604 | 2.116863 |
| GO_REGULATION_OF_CELL_CELL_ADHESION | 0.34930253 | 2.110767 |
| GSE24142_DN2_VS_DN3_THYMOCYTE_FETAL_UP | 0.36910757 | 2.1102414 |
| GSE39820_CTRL_VS_IL1B_IL6_CD4_TCELL_DN | 0.3885687 | 2.1086426 |
| GO_EXTRACELLULAR_MATRIX_COMPONENT | 0.47035378 | 2.1074808 |
| GO_REGULATION_OF_FATTY_ACID_METABOLIC_PROCESS | 0.46695387 | 2.1064765 |
| GO_CELLULAR_RESPONSE_TO_STEROID_HORMONE_STIMULUS | 0.40330642 | 2.1033876 |
| LABBE_TGFB1_TARGETS_UP | 0.45027825 | 2.1024427 |
| GSE43955_1H_VS_60H_ACT_CD4_TCELL_UP | 0.39228988 | 2.09938 |
| SENESE_HDAC1_TARGETS_UP | 0.30395484 | 2.096042 |
| GO_REGULATION_OF_ENDOCYTOSIS | 0.42647433 | 2.0952938 |
| GSE42021_TREG_VS_TCONV_PLN_UP | 0.3595659 | 2.0948052 |
| GSE14699_DELETIONAL_TOLERANCE_VS_ACTIVATED_CD8_TCELL_DN | 0.3824387 | 2.0931172 |
| HIRSCH_CELLULAR_TRANSFORMATION_SIGNATURE_DN | 0.39253253 | 2.0926614 |
| GSE42021_TREG_PLN_VS_TREG_PRECURSORS_THYMUS_DN | 0.36491618 | 2.0914693 |
| GO_NEGATIVE_REGULATION_OF_CATALYTIC_ACTIVITY | 0.29042792 | 2.090259 |
| GSE557_WT_VS_I_AB_KO_DC_DN | 0.38833588 | 2.0878088 |
| GO_REGULATION_OF_EMBRYONIC_DEVELOPMENT | 0.49976107 | 2.0874217 |
| HINATA_NFKB_TARGETS_FIBROBLAST_UP | 0.5372115 | 2.0866535 |
| GO_REGULATION_OF_PEPTIDASE_ACTIVITY | 0.33934164 | 2.0864952 |
| SCHLESINGER_METHYLATED_DE_NOVO_IN_CANCER | 0.5212882 | 2.0857222 |
| MODULE_122 | 0.4855687 | 2.084301 |
| GSE11057_NAIVE_VS_EFF_MEMORY_CD4_TCELL_DN | 0.355042 | 2.0824468 |
| KEGG_NATURAL_KILLER_CELL_MEDIATED_CYTOTOXICITY | 0.46318227 | 2.0793624 |
| GO_RESPONSE_TO_TYPE_I_INTERFERON | 0.51350677 | 2.0789802 |
| PHONG_TNF_RESPONSE_VIA_P38_PARTIAL | 0.36186862 | 2.0722718 |
| GSE11057_EFF_MEM_VS_CENT_MEM_CD4_TCELL_UP | 0.3768663 | 2.0684671 |
| MODULE_64 | 0.34946302 | 2.065733 |
| GSE13485_DAY1_VS_DAY7_YF17D_VACCINE_PBMC_DN | 0.37082955 | 2.0653346 |
| GSE29949_CD8_NEG_DC_SPLEEN_VS_CD8_POS_DC_SPLEEN_DN | 0.41118383 | 2.06451 |
| ALTEMEIER_RESPONSE_TO_LPS_WITH_MECHANICAL_VENTILATION | 0.42954665 | 2.0644104 |
| REACTOME_INTEGRIN_CELL_SURFACE_INTERACTIONS | 0.54748267 | 2.0632532 |
| GO_REGULATION_OF_HOMOTYPIC_CELL_CELL_ADHESION | 0.35641974 | 2.0618753 |
| GSE21670_UNTREATED_VS_IL6_TREATED_STAT3_KO_CD4_TCELL_UP | 0.36952278 | 2.0593994 |
| GSE17721_CTRL_VS_LPS_12H_BMDC_DN | 0.41078824 | 2.0592244 |
| KRAS.600_UP.V1_UP | 0.47578537 | 2.0591915 |
| PURBEY_TARGETS_OF_CTBP1_NOT_SATB1_UP | 0.3331421 | 2.0587475 |
| GO_MICROTUBULE_BASED_PROCESS | 0.30709097 | 2.0586524 |
| GO_PROTEASE_BINDING | 0.46157238 | 2.0519197 |
| MODULE_27 | 0.39313194 | 2.0518577 |
| GSE10239_NAIVE_VS_KLRG1HIGH_EFF_CD8_TCELL_DN | 0.34569588 | 2.0507743 |
| KOYAMA_SEMA3B_TARGETS_UP | 0.33177304 | 2.049242 |
| GOLDRATH_NAIVE_VS_EFF_CD8_TCELL_DN | 0.36806697 | 2.0484474 |
| NABA_ECM_REGULATORS | 0.4407974 | 2.0462906 |
| CSR_LATE_UP.V1_UP | 0.39527807 | 2.0430188 |
| GO_POSITIVE_REGULATION_OF_CELL_CELL_ADHESION | 0.38294217 | 2.0420148 |
| RICKMAN_TUMOR_DIFFERENTIATED_WELL_VS_MODERATELY_DN | 0.46322632 | 2.0401888 |
| GO_REGULATION_OF_LEUKOCYTE_MIGRATION | 0.45240057 | 2.0384781 |
| LIU_PROSTATE_CANCER_DN | 0.30422935 | 2.0384135 |
| P53_DN.V2_UP | 0.52383745 | 2.0384123 |
| GSE9650_NAIVE_VS_MEMORY_CD8_TCELL_DN | 0.347851 | 2.0383565 |
| BASSO_CD40_SIGNALING_UP | 0.4548927 | 2.0380585 |
| TONKS_TARGETS_OF_RUNX1_RUNX1T1_FUSION_ERYTHROCYTE_UP | 0.36758295 | 2.037291 |
| GO_MICROTUBULE | 0.31295648 | 2.034069 |
| GSE32164_ALTERNATIVELY_ACT_M2_VS_CMYC_INHIBITED_MACROPHAGE_DN | 0.38363335 | 2.0336938 |
| GSE29618_PDC_VS_MDC_DN | 0.34443486 | 2.0336761 |
| GSE2770_IL12_AND_TGFB_VS_IL4_TREATED_ACT_CD4_TCELL_48H_DN | 0.41198927 | 2.0291226 |
| SEKI_INFLAMMATORY_RESPONSE_LPS_UP | 0.5140743 | 2.028937 |
| GO_PLASMA_MEMBRANE_RAFT | 0.4335581 | 2.0283697 |
| GO_STRUCTURAL_CONSTITUENT_OF_CYTOSKELETON | 0.44634128 | 2.0279865 |
| GSE5589_WT_VS_IL6_KO_LPS_AND_IL6_STIM_MACROPHAGE_45MIN_DN | 0.35759136 | 2.027375 |
| GO_REGULATION_OF_ACTIN_FILAMENT_BUNDLE_ASSEMBLY | 0.4999497 | 2.0272007 |
| GSE22886_DAY0_VS_DAY1_MONOCYTE_IN_CULTURE_DN | 0.38238224 | 2.023803 |
| GO_CYTOKINE_MEDIATED_SIGNALING_PATHWAY | 0.33484888 | 2.0226398 |
| GSE24026_PD1_LIGATION_VS_CTRL_IN_ACT_TCELL_LINE_UP | 0.3651753 | 2.0209832 |
| GO_NEGATIVE_REGULATION_OF_CELL_ACTIVATION | 0.42119548 | 2.0205061 |
| GO_EXTRACELLULAR_MATRIX_BINDING | 0.52047014 | 2.0179923 |
| PID_CXCR4_PATHWAY | 0.45677334 | 2.0166075 |
| GSE24634_TEFF_VS_TCONV_DAY10_IN_CULTURE_UP | 0.37722328 | 2.0144386 |
| IZADPANAH_STEM_CELL_ADIPOSE_VS_BONE_UP | 0.40133855 | 2.0115733 |
| CHICAS_RB1_TARGETS_SENESCENT | 0.28960317 | 2.010118 |
| ST_INTEGRIN_SIGNALING_PATHWAY | 0.43001354 | 2.0097506 |
| ZHENG_GLIOBLASTOMA_PLASTICITY_UP | 0.33444977 | 2.0064256 |
| GO_ENZYME_INHIBITOR_ACTIVITY | 0.34932697 | 2.0048714 |
| GSE19772_CTRL_VS_HCMV_INF_MONOCYTES_UP | 0.37340853 | 2.0022697 |
| GSE2585_THYMIC_DC_VS_MTEC_DN | 0.38471475 | 2.0013084 |
| LAIHO_COLORECTAL_CANCER_SERRATED_UP | 0.42719015 | 1.9994882 |
| RODRIGUES_THYROID_CARCINOMA_ANAPLASTIC_UP | 0.2677047 | 1.9952707 |
| GSE16385_ROSIGLITAZONE_IL4_VS_IFNG_TNF_STIM_MACROPHAGE_DN | 0.43903795 | 1.9950713 |
| GSE25088_WT_VS_STAT6_KO_MACROPHAGE_IL4_STIM_DN | 0.3897329 | 1.9948144 |
| GSE3565_CTRL_VS_LPS_INJECTED_SPLENOCYTES_UP | 0.38360173 | 1.994609 |
| GO_NEGATIVE_REGULATION_OF_CELL_SUBSTRATE_ADHESION | 0.5312707 | 1.9940661 |
| GSE42021_CD24HI_VS_CD24INT_TREG_THYMUS_DN | 0.35813588 | 1.9935074 |
| WANG_SMARCE1_TARGETS_DN | 0.3061838 | 1.9913105 |
| GO_FORMATION_OF_PRIMARY_GERM_LAYER | 0.44352135 | 1.9910879 |
| GO_GLYCOPROTEIN_BINDING | 0.4765802 | 1.9902618 |
| GO_DNA_GEOMETRIC_CHANGE | 0.47299996 | 1.9847747 |
| GSE24634_TEFF_VS_TCONV_DAY7_IN_CULTURE_DN | 0.37373438 | 1.9821613 |
| GO_REGULATION_OF_MICROTUBULE_BASED_PROCESS | 0.36234888 | 1.9821297 |
| GSE9988_LOW_LPS_VS_ANTI_TREM1_AND_LPS_MONOCYTE_DN | 0.3476583 | 1.9819955 |
| GO_NEGATIVE_REGULATION_OF_MAP_KINASE_ACTIVITY | 0.50266623 | 1.9816499 |
| GSE42021_TREG_PLN_VS_CD24INT_TREG_THYMUS_DN | 0.34705493 | 1.9810084 |
| LINDGREN_BLADDER_CANCER_HIGH_RECURRENCE | 0.50271964 | 1.980172 |
| GO_NEGATIVE_REGULATION_OF_HYDROLASE_ACTIVITY | 0.34209764 | 1.9798559 |
| GSE22935_WT_VS_MYD88_KO_MACROPHAGE_48H_MBOVIS_BCG_STIM_DN | 0.34697193 | 1.9794805 |
| GSE28737_WT_VS_BCL6_HET_MARGINAL_ZONE_BCELL_DN | 0.36661717 | 1.9782459 |
| GSE25123_CTRL_VS_ROSIGLITAZONE_STIM_PPARG_KO_MACROPHAGE_UP | 0.37026134 | 1.9779048 |
| JAATINEN_HEMATOPOIETIC_STEM_CELL_DN | 0.3996833 | 1.9771398 |
| PEDERSEN_METASTASIS_BY_ERBB2_ISOFORM_7 | 0.27438462 | 1.9771289 |
| GO_EXTRINSIC_COMPONENT_OF_MEMBRANE | 0.35276732 | 1.9763811 |
| KEGG_PATHOGENIC_ESCHERICHIA_COLI_INFECTION | 0.50167745 | 1.975352 |
| GSE33513_TCF7_KO_VS_HET_EARLY_THYMIC_PROGENITOR_UP | 0.3917927 | 1.9751701 |
| GO_NEGATIVE_REGULATION_OF_KINASE_ACTIVITY | 0.3490557 | 1.9750351 |
| LEF1_UP.V1_UP | 0.3904038 | 1.9740351 |
| GO_CELL_JUNCTION_ASSEMBLY | 0.41099858 | 1.9732312 |
| ZHU_CMV_ALL_UP | 0.40354592 | 1.9698424 |
| VECCHI_GASTRIC_CANCER_ADVANCED_VS_EARLY_UP | 0.4062825 | 1.9674054 |
| MODULE_223 | 0.40952405 | 1.9662898 |
| GSE28726_ACT_CD4_TCELL_VS_ACT_NKTCELL_UP | 0.35025814 | 1.9661753 |
| HUANG_GATA2_TARGETS_UP | 0.39493054 | 1.9625823 |
| GO_REGULATION_OF_RECEPTOR_ACTIVITY | 0.41351107 | 1.9622781 |
| GO_MEMBRANE_DOCKING | 0.5303965 | 1.9611017 |
| CHIANG_LIVER_CANCER_SUBCLASS_CTNNB1_DN | 0.37137195 | 1.9603993 |
| GO_CELL_MATRIX_ADHESION | 0.46367702 | 1.9595152 |
| PID_TAP63_PATHWAY | 0.43761152 | 1.9591638 |
| DELYS_THYROID_CANCER_UP | 0.2787454 | 1.9547437 |
| KERLEY_RESPONSE_TO_CISPLATIN_UP | 0.45379958 | 1.9543523 |
| ZHU_CMV_24_HR_UP | 0.4378316 | 1.9537295 |
| GSE39110_UNTREATED_VS_IL2_TREATED_CD8_TCELL_DAY6_POST_IMMUNIZATION_UP | 0.3683142 | 1.9537047 |
| GSE36826_WT_VS_IL1R_KO_SKIN_DN | 0.34452686 | 1.9524456 |

**Figure S3**


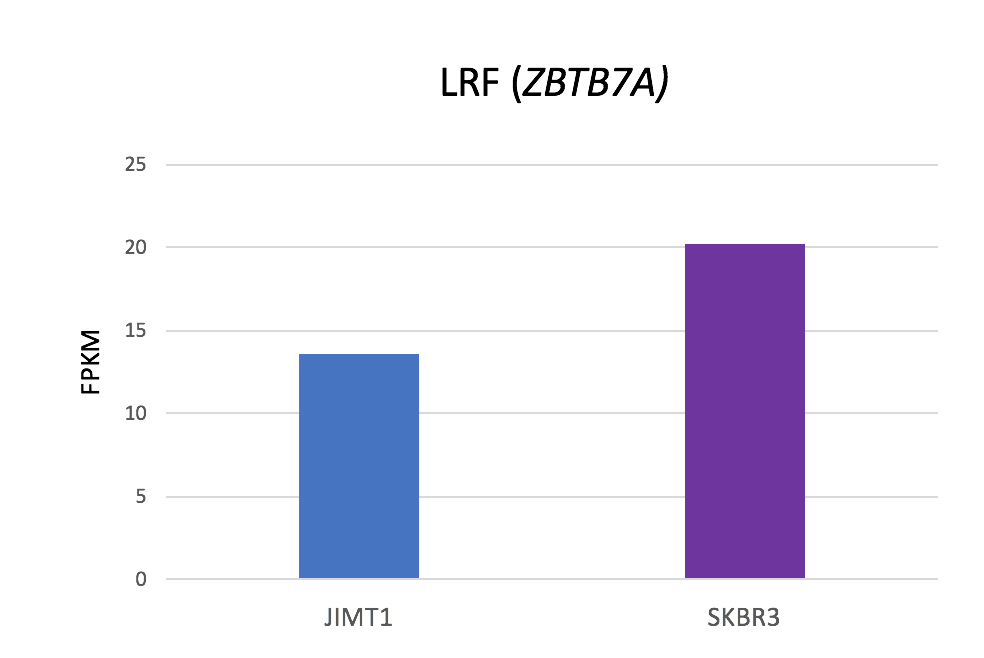


**Figure S4**


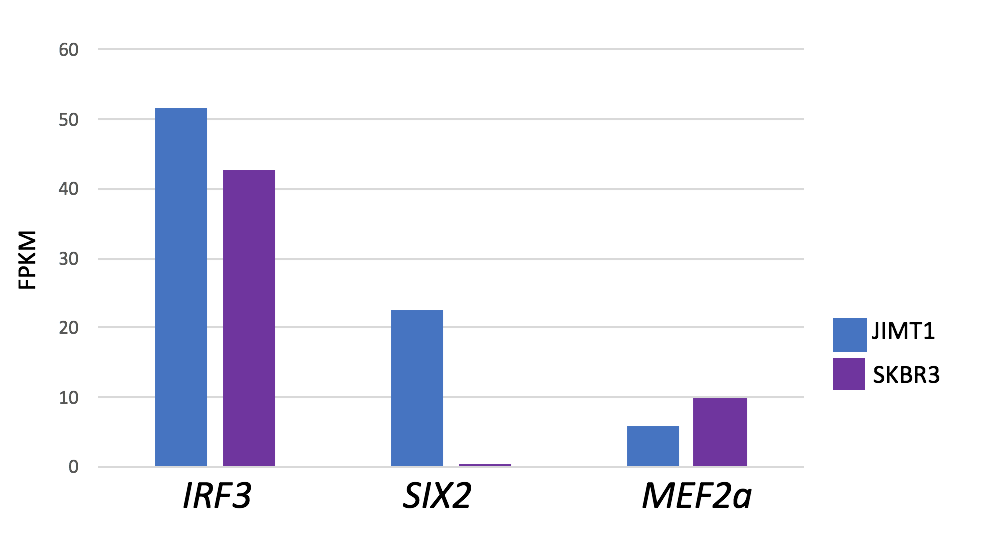


**Figure S5**

**
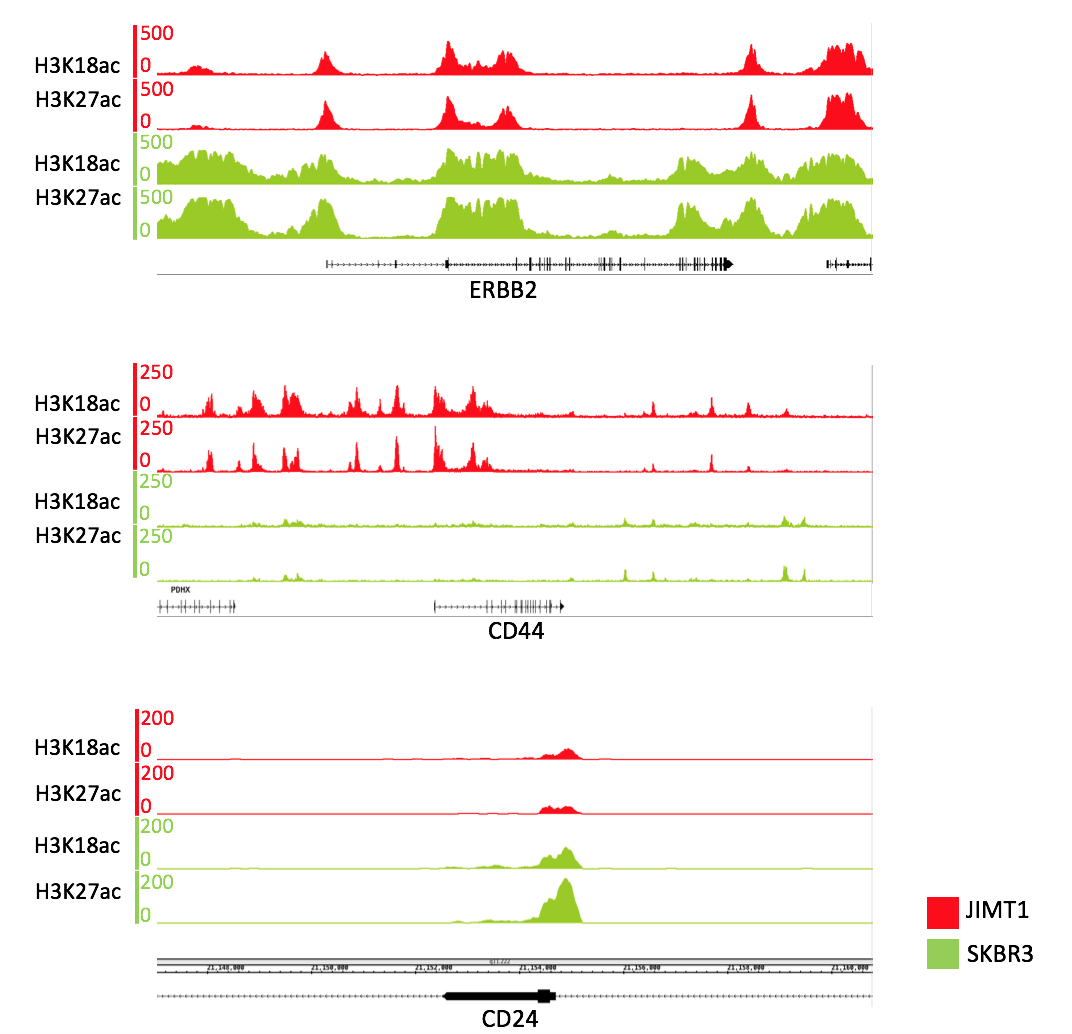
**

**Figure S6**

**
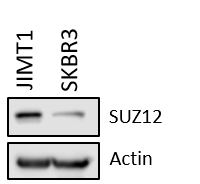
**

**Figure S7**


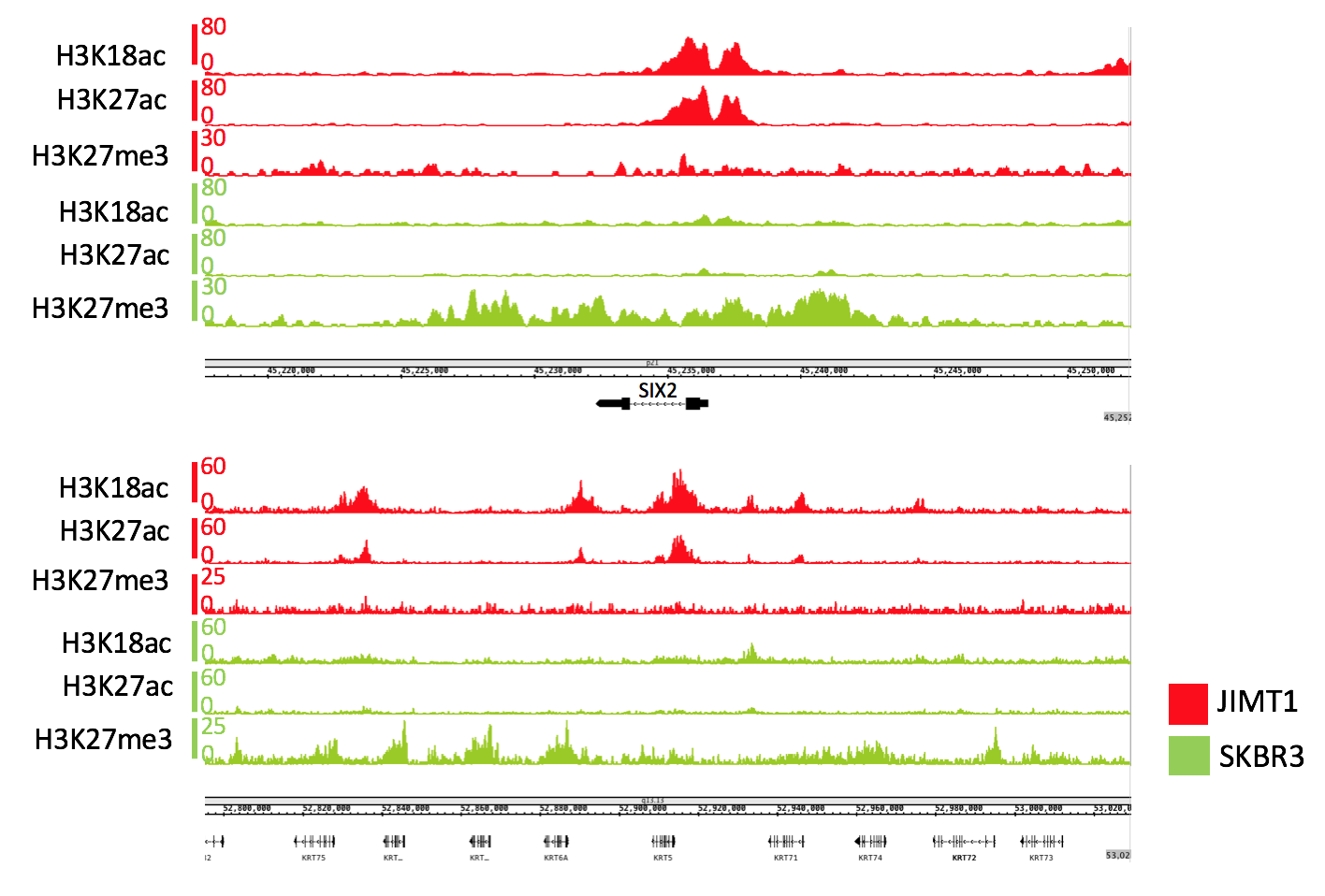


**Figure S8
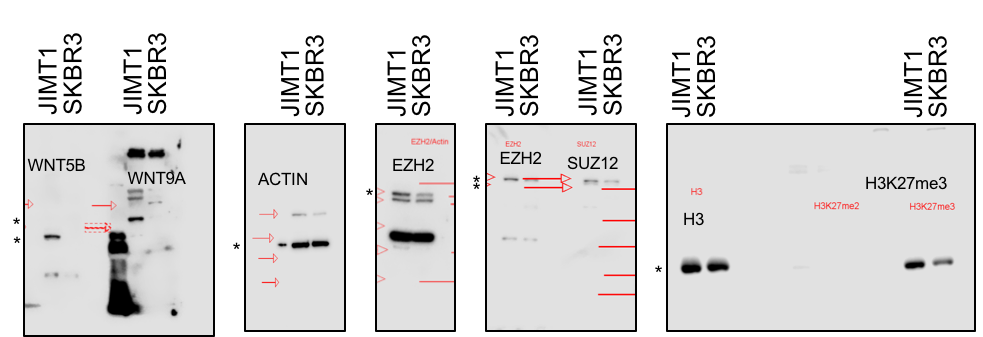
**
